# DCAF7/WDR68 is required for normal levels of DYRK1A and DYRK1B

**DOI:** 10.1371/journal.pone.0207779

**Published:** 2018-11-29

**Authors:** Mina Yousefelahiyeh, Jingyi Xu, Estibaliz Alvarado, Yang Yu, David Salven, Robert M. Nissen

**Affiliations:** Department of Biological Sciences, California State University Los Angeles, Los Angeles, California, United States of America; Wayne State University, UNITED STATES

## Abstract

Overexpression of the Dual-specificity Tyrosine Phosphorylation-Regulated Kinase 1A (*DYRK1A*) gene contributes to the retardation, craniofacial anomalies, cognitive impairment, and learning and memory deficits associated with Down Syndrome (DS). DCAF7/HAN11/WDR68 (hereafter WDR68) binds DYRK1A and is required for craniofacial development. Accumulating evidence suggests DYRK1A-WDR68 complexes enable proper growth and patterning of multiple organ systems and suppress inappropriate cell growth/transformation by regulating the balance between proliferation and differentiation in multiple cellular contexts. Here we report, using engineered mouse C2C12 and human HeLa cell lines, that WDR68 is required for normal levels of DYRK1A. However, *Wdr68* does not significantly regulate *Dyrk1a* mRNA expression levels and proteasome inhibition did not restore DYRK1A in cells lacking *Wdr68* (Δwdr68 cells). Overexpression of WDR68 increased DYRK1A levels while overexpression of DYRK1A had no effect on WDR68 levels. We further report that WDR68 is similarly required for normal levels of the closely related DYRK1B kinase and that both DYRK1A and DYRK1B are essential for the transition from proliferation to differentiation in C2C12 cells. These findings reveal an additional role of WDR68 in DYRK1A-WDR68 and DYRK1B-WDR68 complexes.

## Introduction

Birth defects are among the leading causes of infant mortality. Cleft lip with or without cleft palate (CL/P) affects 1 in 589 births [1]. Many craniofacial syndromes are caused by defects in signaling pathways. For example, the *DCAF7/HAN11/WDR68* (hereafter *WDR68*) gene is linked to CL/P [2] and required for Endothelin-1 (EDN1) signaling [3]. Defects in EDN1 signaling cause Auriculocondylar syndrome [4–6]. Down Syndrome (DS) affects 1 in 691 births [1]. Overexpression of the Dual-specificity Tyrosine Phosphorylation-Regulated Kinase 1A (*DYRK1A*) gene contributes to the retardation, cognitive impairment, and learning and memory deficits associated with DS [7–10]. Conversely, human *DYRK1A* haploinsufficiency causes microcephaly [11–13]. In mice, *Dyrk1a* knock-out embryos are severely reduced by E9.5 and die by E11.5 [14]. WDR68 binds DYRK1A [3, 15, 16], and this interaction is important for substrate recruitment [17]. WDR68 can also regulate the activity of certain kinases [18], and the interaction between WDR68 and DYRK1A is subject to regulation [19]. Nonetheless, how WDR68 binding impacts partner kinase functions remains incomplete.

WD40 repeat domain-containing proteins function as scaffolding elements for the assembly of multi-subunit protein complexes [20]. Originally identified in plants for a role in anthocyanin biosynthesis [21], WDR68 is a 342 amino acid length protein composed of five WD40 repeats that modeling suggests forms a seven-blade ß-propeller structure [22]. In zebrafish, Wdr68 is important for embryonic development of the upper and lower jaws [3, 23–25]. WDR68 has also been identified as a DDB1 and CUL4-associated factor (DCAF), thus implicating it in the ubiquitin-mediated regulation of protein stability [26]. WDR68 binds and mediates the ubiquitin-dependent destruction of DNA Ligase I [27].

DYRK1A is an important regulator of the balance between cell proliferation and differentiation (reviewed in [28–32]). In *Drosophila*, the *DYRK1A* ortholog *minibrain* is important for proper size of the central brain hemispheres [33]. In mice, *Dyrk1a* haploinsufficiency likewise yields smaller pups with reduced brain size [14]. DYRK1A levels are dynamic in the brain and it shuttles between cytoplasmic and nuclear compartments across key developmental transitions [34–36]. DYRK1A enables the acquisition of competence for neuronal differentiation [37]. DYRK1A promotes cell cycle exit and quiescence by facilitating DREAM complex assembly via phosphorylation of LIN52 [38, 39]. Likewise, DYRK1A overexpression can inhibit cell proliferation and induce premature neuronal differentiation by phosphorylating p27^**kip1**^ and CYCLIN D1 [40]. DYRK1A also functions as a nuclear transcriptional co-activator via phosphorylation of the RNApII-CTD [41], and recruitment of histone acetyl transferases [42], to regulate a variety of genes implicated in cell cycle control.

The DYRK1A-WDR68 complex was first identified by biochemical purification of an approximately 138kD complex capable of phosphorylating GSK3 [15]. The DYRK1A-WDR68 complex is largely found within the cell nucleus [3, 16, 18, 25]. This nuclear localization is driven by an NLS in DYRK1A that is nearby, but distinct from, the WDR68 binding site in DYRK1A. Notably, the WDR68 binding site is highly conserved across DYRK1A orthologs ranging from yeast to mammals [17]. In adenovirus and HPV infection models, WDR68 facilitates substrate recruitment to DYRK1A [17] to suppress cell growth and transformation via phosphorylation of the E1A and E6 proteins, respectively [43–46]. In *Drosophila*, the genes orthologous to *Dyrk1a* (*minibrain*) and *Wdr68* (*wings apart*) similarly interact to phosphorylate and inhibit the transcriptional repressor *capicua* thereby enabling proper growth and patterning of several organ systems [47, 48].

Together these previous findings support a model in which a DYRK1A-WDR68 complex regulates the balance between proliferation and differentiation in multiple contexts ranging from embryonic to adult life stages. Here we report further refinements to the model of DYRK1A-WDR68 interaction. Specifically, we found that WDR68 is required for normal DYRK1A protein levels in both mouse and human cells, that *Wdr68* does not regulate *Dyrk1a* mRNA expression or stability, and that proteasome inhibition does not restore DYRK1A levels in cells lacking WDR68 (Δwdr68 cells). We also report that the requirement of WDR68 for DYRK1A is unidirectional because the level of WDR68 is not affected in cells lacking DYRK1A (Δdyrk1a cells). Consistently, we report that overexpression of WDR68 increases DYRK1A levels while overexpression of DYRK1A had no effect on WDR68 levels. Furthermore, we report that WDR68 is similarly required for normal levels of the closely related DYRK1B kinase and that both DYRK1A and DYRK1B are essential for the transition from proliferation to differentiation in C2C12 cells. These new findings improve our models of DYRK1A-WDR68 and DYRK1B-WDR68 complex function while extending prior observations on DYRK1A and DYRK1B as critical regulators of the balance between cell proliferation and differentiation. The fact that WDR68 protein level directly correlates with the level of DYRK1A suggests that manipulating the levels of WDR68 could be explored as a potential new avenue for treating DS.

## Materials and methods

### Chemicals and reagents

C2C12 cells and HeLa cells were obtained from the ATCC and cultured in growth medium (GM) (DMEM (100013CV, Corning); 15% FBS (35015CV, Corning); 4.8 mM L-glutamine (1680149, MP Biomedicals); 100μg/mL pen/strep (SV30010, GE Healthcare)). Cells were passaged using 0.25% Trypsin, 2.21mM EDTA, 1X (25-053-CI, Corning) and Phosphate Buffered Saline 1X (PBS) (MT1040CM, Corning). Reagents used for protein extracts and western blots were Halt Protease Inhibitor Cocktail 100X (PIC) (1860932, Thermo Fisher Scientific), Pierce BCA Protein Assay Kit (23227, Thermo Fisher Scientific), PVDF membrane (88518, Thermo Fisher Scientific), Novex WedgeWell 8–16% Tris-Glycine Gel (XP08162BOX, Thermo Fisher Scientific), Amersham ECL Western Blotting Analysis System (RPN2108, GE healthcare), N-Ethylmaleimide (NEM) (128-53-0, Thermo Fisher Scientific), HEPES (7365-45-9, Thermo Fisher Scientific), DL-1, 4-Dithiothreitol (DTT) (3483-12-3, Thermo Fisher Scientific), CaCl_2_ (10035-04-8, Fisher Scientific), Calpain-Glo Protease Assay (G8501, Promega). Antibodies used were anti-WDR68 (HPA022948, Sigma-Aldrich), anti-DYRK1A (8765S, Cell Signaling), anti-DYRK1B (5672S, Cell Signaling), anti-Myogenin (sc-52903, Santa Cruz Biotechnology), anti-Ubiquitin (3933s, Cell Signaling), anti-β-tubulin (sc-55529, Santa Cruz Biotechnology Inc.), goat anti-mouse IgG-HRP (sc-2005, Santa Cruz Biotechnology Inc.), anti-rabbit IgG, HRP-linked whole antibody (from donkey) (NA934, GE Healthcare). Drugs used were G418 (ant-gn-1, InvivoGen), puromycin (P9620, Sigma-Aldrich), LLNL (A6185, Sigma-Aldrich), Epoxomicin (A2606, ApexBio), MG132 (133407-82-6, Cayman Chemical), Chloroquine Diphosphate (CQ) (50-63-5, Thermo Fisher Scientific).

### Western blots, drug treatments, and immunofluorescence

Western blots were performed as previously described [23, 25]. Briefly, cell extracts were made from 10cm or 6-well plates of cells in GM or Differentiation Medium (DM) (DMEM; 2% Horse serum; 100ug/mL pen/strep). HeLa cell extracts were made from 10cm plates or 6-well plates of cells in GM. Cells were rinsed twice with ice-cold PBS, and then incubated with ice-cold RIPA buffer (50mM tris-HCl, 150mM NaCl, 1% Igepal-CA630, 0.5% Na Deoxycholate, 0.1% SDS, 1x PIC) for 5 minutes at 4°C. Cells were then scraped from the plate, shake for 15 minutes at 4°C, centrifuged at 10,000xg for 10 minutes at 4°C and supernatants quantified by BCA assay prior to being subjected to western blot analysis as follows. 20μg of each protein samples with SDS-PAGE loading buffer were boiled for 5 minutes at 95°C, ran on 8–16% SDS-PAGE gels, and then transferred onto PVDF membrane. The PVDF membrane was blocked 1 hour at room temperature with 5% non-fat dry milk in TBST (1X TBS+0.1% Tween-20) with 0.01% NaN_3_. The following day the blocking buffer was removed and blocked primary antibody was added. The membrane was then rinsed three times for 15 minutes with TBST and then secondary antibody was added. After 2 hours, the membrane was rinsed three times for 15 minutes with TBST and imaged by Versadoc (Bio-Rad).

Images of western blots were quantified using ImageJ for bands of interest and then plotted for quantitative analysis in Microsoft Excel. Mean values and standard deviations for each protein were calculated from at least three biological replicates. Significance was calculated by by one-way ANOVA and post-hoc Tukey HSD with p<0.05 as the threshold for significance.

For drug treatments, cells were exposed to DMSO vehicle, 50μM LLNL for 6 hours, 50μM epoxomicin for 6 hours, 50μM MG132 for 8 hours, or 12.5 μM CQ for 8 hours and then harvested for extracts in RIPA supplemented with 1% NEM and 1mM EDTA.

For calpain activity assay after LLNL treatment, cells were harvested for extracts in RIPA (without PIC) supplemented with 1% NEM and 1mM EDTA. Extracts were activated by 10mM HEPES, 10mM DTT, 10mM EDTA, and 2mM CaCl_2_, and then mixed with Calpain-Glo buffer, luciferin detection reagent, and Suc-LLVY-Glo substrate from Calpain-Glo Protease Assay according to the manufacturer’s protocol. The luminescence was read by a microplate luminometer. Immunoflourescence was performed as previously described [25].

### Isolation of CRISPR/Cas9-mediated gene disruptions

The C2C12 NT1 and Δwdr68 cells were previously described [23]. Additional sublines were generated as previously described [23, 49]. Briefly, pLentiCRISPRv2-mdyrk1a1 was generated by annealing the oligonucleotides CRISPR-mdyrk1a-1f: 5’-CACCGTTGCGCAAACTTTCGTGTT-3’ and CRISPR-mdyrk1a-1r: 5’-AAACAACACGAAAGTTTGCGCAAC-3’. pLentiCRISPRv2-mdyrk1a2 was generated by annealing the oligonucleotides CRISPR-mdyrk1a-2f: 5’- CACCGATCTTGATTGCACTCCGTTT-3’ and CRISPR-mdyrk1a-2r: 5’- AAACAAACGGAGTGCAATCAAGATC-3’. pLentiCRISPRv2-mdyrk1b1 was generated by annealing the oligonucleotides CRISPR-mdyrk1b-1f: 5’-CACCGTGTTGCGGAGGAGGTCGTAC-3’ and CRISPR-mdyrk1b-1r: 5’-AAACGTACGACCTCCTCCGCAACAC-3’. pLentiCRISPRv2-mdyrk1b2 was generated by annealing the oligonucleotides CRISPR-mdyrk1b-2f: 5’-CACCGGAACATGAAGTGCCGCTTA-3’ and CRISPR-mdyrk1b-2r: 5’-AAACTAAGCGGCACTTCATGTTCC-3’. pLentiCRISPRv2-dcaf7-1 was generated by annealing the oligonucleotides CRISPR-dcaf7-1f: 5’- CACCGCGGTGACTATCTCCGTGTG-3’ and CRISPR-dcaf7-1r: 5’-AAACCACACGGAGATAGTCACCGC-3’. pLentiCRISPRv2-dcaf7-2 was generated by annealing the oligonucleotides CRISPR-dcaf7-2f: 5’- CACCGGTGGGGTATGGGTGGTCAA-3’ and CRISPR-dcaf7-2r: 5’- AAACTTGACCACCCATACCCCACC-3’. Annealed oligonucleotides were then ligated into the BsmBI-digested pLentiCRISPRv2 vector fragment, transformed into Stbl3 competent cells, clones isolated and verified by DNA sequencing. Lentiviral particles were generated by co-transfecting 293T cells with the virus packaging plasmids psPAX2 and pCMV-VSV-G along with the various pLentiCRISPR derivatives. Cells were then transduced with cleared virus-containing supernatant in appropriate growth medium, followed by puromycin selection, subclone isolation by serial dilution, screening by Western blot, mutant allele cloning, and DNA sequencing. Cell lines are available upon request.

### Isolation of siRNA knockdown subline

Transduction of C2C12 cells with *Wdr68*-siRNA constructs was previously described [25]. Subclones were isolated by serial dilution under puromycin selection and then screened by anti-WDR68 Western blot to identify subline siWdr68-4650-1. Cell lines are available upon request.

### Transient transfections and reporter assays

The pG5D1AISLAND-LUC (D1A) plasmid was previously described [41]. The luciferase and SV-40 Renilla plasmids were co-transfected into cells using X-tremeGENE HP DNA Transfection Reagent (6366244001, Sigma-Aldrich) per the manufacturer‘s recommendations. DNA-lipid complexes were replaced with fresh medium 16 hours later. After an additional 8 hours, cell extracts were harvested for luminometer measurements using the Dual-luciferase reporter (DLR) assay kit reagents (E1910, Promega). Four independent biological replicates of the transient transfection, each containing three technical replicates, were analyzed. Data were analyzed by one-way ANOVA and post-hoc Tukey HSD with p<0.05 as the threshold for significance.

For western blots, cells were transiently transfected with pEGFPC2, pEGFPC2-Wdr68 [25] or pEGFP-Dyrk1a [50] for 24hrs, medium refreshed for 24hrs, and then harvested for extracts.

### Reverse transcription qPCR

RNA was isolated from cells using TRIzol Reagent following manufacturer recommendations (15596026, Invitrogen). First-Strand cDNA synthesis was done according to manufacturer instructions using SuperScript II Reverse Transcriptase (18064014, Thermo Fisher Scientific) and oligo-dT primer. 2μL of cDNA was used as template in PCR reactions using the following primers: mWdr68-f2: 5’-ACCTCCGCCATCTGGAACATAGCAC-3’ and mWdr68-r2: 5’-ACGTGAGGTGCCACCAACACTACAC-3’; mDyrk1A-f3: 5’-GTGGATCCTCGGGAACGAGT-3’ and mDyrk1a-r3: 5’-CGGTTACCCAAGGCTTGCTG-3’; mDyrk1b-f4: 5’-CTACGGCTGTTGGAGCTGATGAA-3’ and mDyrk1b-r4: 5’-TGGTCCACCTCATTAGAGCCACT-3’; reference gene for normalization to 18Sreg3F: 5’-ctcaacacgggaaacctcac-3’ and 18Sreg3R: 5’-cgctccaccaactaagaacg-3’ and GoTaq qPCR Master Mix (A6001, Promega) on a Eppendorf RealPlex2. Three biological replicates were analyzed using a modified ΔΔCt method [51], relative to 18S rRNA reference with one-way ANOVA and post-hoc Tukey HSD on ΔCt values with p<0.05 as the threshold for significance, followed by conversion to fold expression.

## Results

### WDR68 is required for cells to reach normal levels of DYRK1A

**Fig 1:**
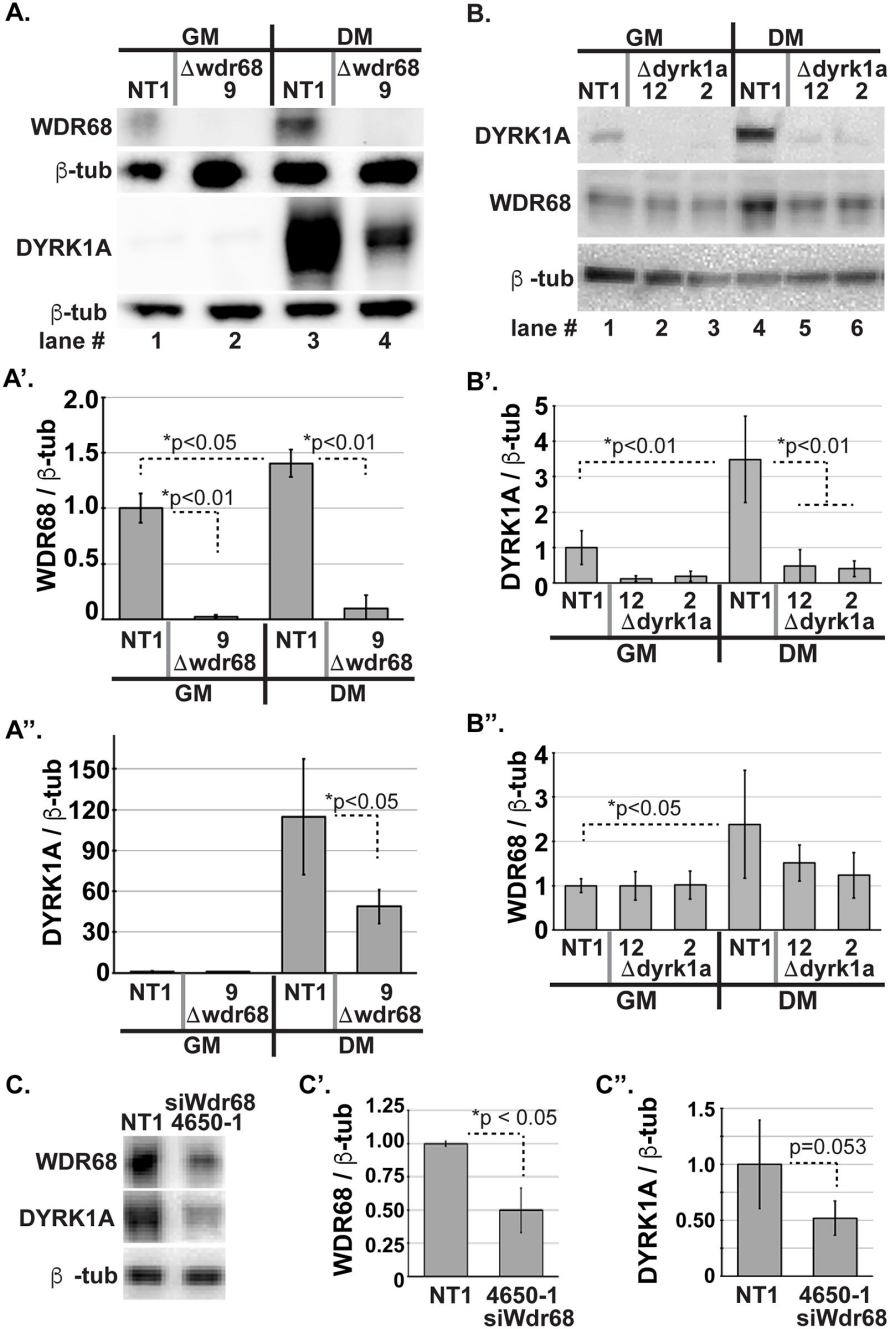
WDR68 is required for normal levels of DYRK1A in C2C12 cells. A) C2C12 NT1 and Δwdr68-9 sublines in growth medium (GM, lanes 1–2) versus differentiation medium (DM, lanes 3–4). WDR68 panel: Lanes 1 and 3, WDR68 was detected in NT1 cells and induced higher in DM. Lanes 2 and 4, WDR68 was not detected above background in Δwdr68-9 cells. DYRK1A panel: Lanes 1 and 3, DYRK1A was detected in NT1 cells and induced higher in DM. Lanes 2 and 4, reduced DYRK1A expression in Δwdr68-9. β-tubulin panel: β-tubulin controls indicated similar loading in each lane. A’) Quantitative analysis confirmed significantly reduced levels of WDR68 in the Δwdr68-9 subline. Three independent biological replicates were analyzed. A”) Quantitative analysis confirmed significantly reduced DYRK1A expression in the Δwdr68-9 subline. Four independent biological replicates were analyzed. B) C2C12 NT1 and Δdyrk1a sublines in GM (lanes 1–3) versus DM (lanes 4–6). DYRK1A panel: Lanes 1 and 4, DYRK1A was detected in NT1 cells and induced higher in DM. Lanes 2 and 5, reduced DYRK1A level in Δdyrk1a-12 cells. Lanes 3 and 6, reduced DYRK1A level in Δdyrk1a-2 cells. WDR68 panel: Lanes 1 to 6, WDR68 was detected in NT1, Δdyrk1a-12, and Δdyrk1a-2 cells. β-tubulin panel: β-tubulin controls indicated similar loading in each lane. B’) Quantitative analysis confirmed significantly reduced DYRK1A level in the Δdyrk1a sublines. Three independent biological replicates were analyzed. B”) Quantitative analysis revealed significant induction of WDR68 in NT1 cells cultured in DM versus GM, but no significant differences in WDR68 levels between NT1, Δdyrk1a-12, and Δdyrk1a-2 cells in DM. Four independent biological replicates were analyzed. C) C2C12 NT1 and *siWdr68*-4650-1 cells in DM. WDR68 panel: Lane 1, WDR68 was detected in NT1 cells. Lane 2, reduced WDR68 level in *siWdr68*-4650-1 cells. DYRK1A panel: Lane 1, DYRK1A was detected in NT1 cells. Lane 2, reduced DYRK1A level in *siWdr68*-4650-1 cells. β-tubulin panel: β-tubulin controls indicated similar loading in each lane. C’). Quantitative analysis confirmed significantly reduced WDR68 level in the *siWdr68*-4650-1 subline. Three independent biological replicates were analyzed. C”). Quantitative analysis confirmed significantly reduced DYRK1A in the *siWdr68*-4650-1 subline. Three independent biological replicates were analyzed. All plots show means +/- standard deviations.

C2C12 cells grow rapidly when cultured in growth medium (GM), but arrest cellular growth and differentiate into multinucleated myoblasts in low-serum/differentiation medium (DM) [52, 53]. WDR68 is important for this developmental transition [25], and we previously generated Δwdr68 mouse C2C12 sublines and non-targeted (NT1) control cells [23]. Upon more careful analysis, we found that the level of WDR68 is significantly induced in DM (Fig 1A and 1A’, compare lane 3 to lane 1, p<0.05). Consistent with our previous report [23], WDR68 is not detected above background in Δwdr68-9 cells relative to NT1 control cells (Fig 1A and 1A’, compare lane 2 to lane 1 and lane 4 to lane 3, p<0.01). Next, we examined the level of DYRK1A in NT1 control cells and similarly found that the level of DYRK1A is significantly higher in DM than GM (Fig 1A and 1A”, compare lane 3 to lane 1, p<0.01). Remarkably, we also found that the level of DYRK1A is significantly reduced in Δwdr68-9 cells relative to NT1 control cells in DM (Fig 1A and 1A”, compare lane 4 to lane 3). We examined the levels of β-tubulin as the loading control and for normalization and found them to be similar (Fig 1A, 1A’ and 1A”, lanes 1–4). Thus, WDR68 is required for mouse C2C12 cells to reach normal levels of DYRK1A.

To determine whether the converse is also true, we used CRISPR/Cas9-mediated mutagenesis to generate Δdyrk1a mouse C2C12 sublines (Fig 1B, S1 Table). We targeted coding exon #5, which encodes part of the kinase domain, of the mouse *Dyrk1a* gene in order to induce frame-shift mutations incapable of yielding full-length functional protein. The Δdyrk1a-2 cells contain a -17 deletion allele and a +1 insertion allele, both of which are predicted to yield frame-shifts incapable of forming full-length protein. The Δdyrk1a-12 cells contain a -22 deletion allele and a -4 deletion allele, both of which are predicted to yield frame-shifts incapable of forming full-length protein. Again, we found that the level of DYRK1A is significantly higher in DM than GM (Fig 1B and 1B’, compare lane 4 to lane 1, p<0.01). As expected given the nature of the mutations, the level of DYRK1A is severely and significantly reduced in Δdyrk1a cells relative to NT1 control cells (Fig 1B and 1B’, compare lanes 5 and 6 to lane 4, p<0.01). In contrast, we found that the level of WDR68 is not significantly reduced in Δdyrk1a cells relative to NT1 controls (Fig 1B and 1B”, compare lanes 5 and 6 to lane 4). We examined the levels of β-tubulin as the loading control and for normalization and found them to be similar (Fig 1B, 1B’ and 1B”, lanes 1–6). Thus, DYRK1A is not required for normal WDR68 protein levels.

To assess whether partial reduction of WDR68 level would yield a partial reduction in DYRK1A level, we isolated a stable siRNA-wdr68 subline (*siWdr68*-4650-1) using constructs previously described [25]. As expected, the level of WDR68 is reduced in *siWdr68*-4650-1 cells relative to NT1 control cells (Fig 1C and 1C’, compare lane 2 to lane 1). We also found that the level of DYRK1A is reduced in *siWdr68*-4650-1 cells relative to NT1 control cells (Fig 1C and 1C”, compare lane 2 to lane 1). We examined the levels of β-tubulin as the loading control and for normalization and found them to be similar (Fig 1C, 1C’ and 1C”, lanes 1–2). Thus, we found that partial reduction of WDR68 yielded a partial reduction of DYRK1A level suggesting a strict positive relationship between the level of WDR68 and the level of DYRK1A.

**Fig 2:**
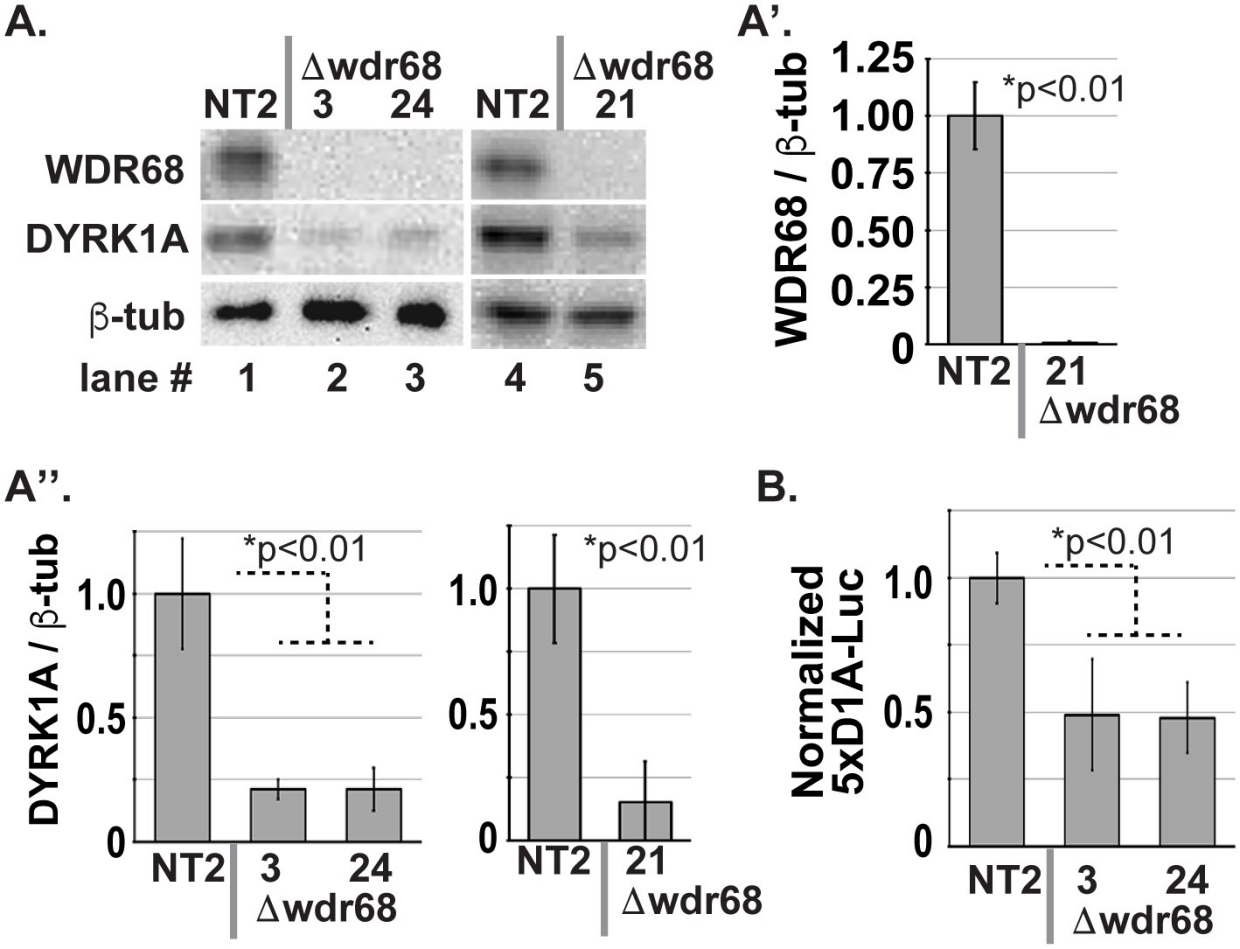
WDR68 is required for normal levels of DYRK1A in HeLa cells. A) HeLa NT2 and Δwdr68 sublines. WDR68 panel: Lanes 1 and 4, WDR68 was detected in NT2 cells. Lanes 2, 3, and 5, WDR68 was not detected above background in Δwdr68-3, Δwdr68-24, and Δwdr68-21 cells. DYRK1A panel: Lanes 1 and 4, DYRK1A was detected in NT2 cells. Lanes 2, 3, and 5, reduced levels of DYRK1A in Δwdr68-3, Δwdr68-24, and Δwdr68-21 cells. β-tubulin panel: β-tubulin controls indicated similar loading in each lane. A’) Quantitative analysis confirmed significantly reduced level of WDR68 in the Δwdr68-21 subline. Three independent biological replicates were analyzed. A”) Quantitative analysis confirmed significantly reduced levels of DYRK1A in the Δwdr68 sublines. Three independent biological replicates were analyzed. B) HeLa NT2 control cells displayed the normal baseline level of 5xDyrk1a response element reporter activity. The Δwdr68-3 and Δwdr68-24 cells displayed 49-/+21% and 48-/+13% relative to NT2 controls (p<0.01). Four independent biological replicates were analyzed. All plots show means +/- standard deviations.

We next sought to determine whether the observed requirement of WDR68 for DYRK1A might somehow be specific only to mouse C2C12 cells, or is the regulatory relationship more general and thus also true in human cells. HeLa cells were derived from a human cervical carcinoma [54], and are the most commonly used cell line. We again used CRISPR/Cas9-mediated mutagenesis to generate Δwdr68 human HeLa sublines as well as a non-targeted (NT2) control subline (Fig 2, S2 Table). We targeted coding exon #2 of the human *DCAF7/WDR68* gene in order to induce frame-shift mutations incapable of yielding full-length functional protein. The Δwdr68-3 cells contain a +1 insertion allele and a +303/-10 insertion/deletion allele, both of which are predicted to yield frame-shifts incapable of forming full-length protein. The Δwdr68-21 cells contain a +1 insertion allele and a +1/-2 insertion/deletion allele, both of which are predicted to yield frame-shifts incapable of forming full-length protein. The Δwdr68-24 cells contain a +3/-2 insertion/deletion allele and a -10 deletion allele, both of which are predicted to yield frame-shifts incapable of forming full-length protein. As expected, WDR68 was not detectable above background in Δwdr68 cells relative to NT2 control cells (Fig 2A compare lanes 2, 3 and 5 to lanes 1 and 4, and Fig 2A’ p<0.01). Likewise, we found that DYRK1A levels are significantly reduced in human Δwdr68 cells relative to NT2 controls (Fig 2A compare lanes 2, 3 and 5 to lanes 1 and 4, and Fig 2A” p<0.01). We examined the levels of β-tubulin as the loading control and for normalization and found them to be similar (Fig 2A). Thus, WDR68 is required for both mouse and human cells to reach normal levels of DYRK1A.

DYRK1A is an RNApII CTD-kinase that localizes to a specific DNA sequence 5’-TCTCGCGAGA-3’ in cell nuclei. DYRK1A likely acts as a nuclear transcriptional co-activator of gene expression by tethering to an as yet unidentified DNA binding protein capable of recognizing that sequence [41]. In humans, *DYRK1A* haploinsufficiency causes microcephaly [11–13], indicating that a 50% reduction in the level of DYRK1A can yield negative functional consequences. Because Δwdr68 cells showed even lower reductions of DYRK1A than that (Figs 1A” and 2A”), we sought to determine whether DYRK1A-mediated co-activation of gene expression is also compromised in Δwdr68 cells. We used the previously described luciferase reporter pG5D1AIsland-Luc (D1A) that harbors five copies of the 5’-TCTCGCGAGA-3’ motif upstream of a minimal promoter [41]. We then transiently transfected it along with SV40-Renilla for normalization in dual-luciferase assays (Fig 2B). We readily detected D1A activity in HeLa NT2 control cells (Fig 2B, column 1). In contrast, D1A activity in human Δwdr68 cells was significantly reduced relative to NT2 controls (Fig 2B, compare columns 2 and 3 to column 1, respectively, p<0.01). Thus, the reduced levels of DYRK1A found in Δwdr68 cells is severe enough to compromise a downstream read-out of a DYRK1A function.

## *Wdr68* is not required for *Dyrk1a* transcript accumulation

**Fig 3:**
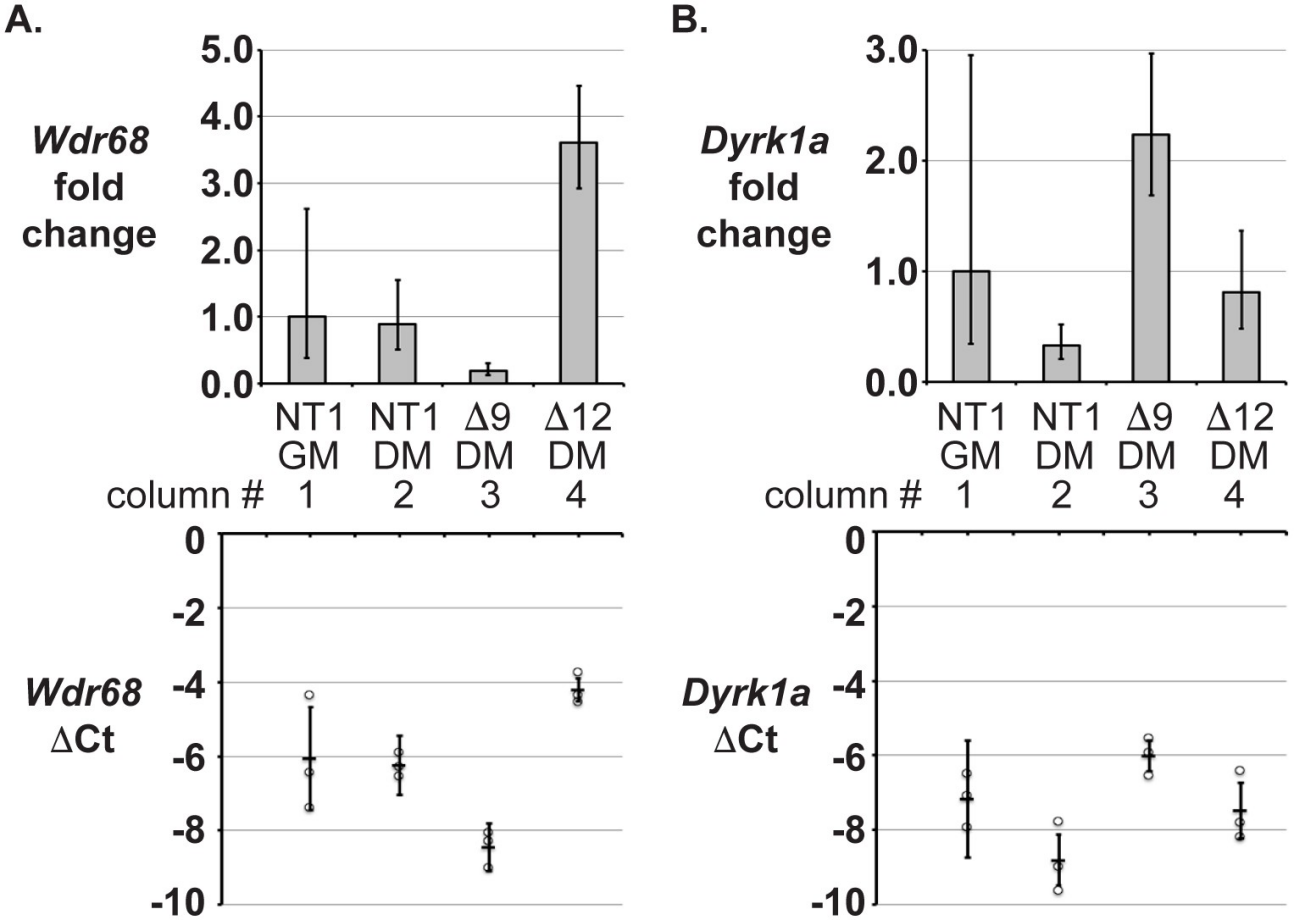
*Wdr68* is not required for *Dyrk1a* mRNA accumulation. A-B) RT-qPCR analysis of C2C12 sublines. Column 1 is NT1 cells in GM. Column 2 is NT1 cells in DM. Column 3 is Δwdr68-9 (Δ9) cells in DM. Column 4 is Δdyrk1a-12 (Δ12) cells in DM. The bottom graph shows the individual ΔCt values (circles) of replicates and the average ΔCt (horizontal bar) -/+ standard deviations. The top graph shows the 2^ΔΔCt^ values (fold change relative to NT1 GM) with corresponding low and high ranges. (A) Analysis of *Wdr68* mRNA expression. B) Analysis of *Dyrk1a* mRNA expression. Three independent biological replicates were analyzed.

The regulation of gene expression is complex and can involve multiple distinct levels of control. To determine whether *Wdr68* is required for *Dyrk1a* mRNA accumulation, we performed RT-qPCR analysis on NT1 control, Δwdr68, and Δdyrk1a cells. *Wdr68* mRNA was readily and similarly detected in NT1 control cells in GM and in DM (Fig 3A, compare column 1 vs 2). As expected, *Wdr68* mRNA expression was reduced in Δwdr68-9 cells to about 20% of that found in NT1 controls (Fig 3A, column 3 vs 2, p = 0.053), likely due to nonsense-mediated decay. *Wdr68* mRNA expression trended upwards in Δdyrk1a-12 cells relative to NT1 controls (Fig 3A, column 4 vs 2, p = 0.077). *Dyrk1a* mRNA was readily and similarly detected in NT1 cells in GM and in DM (Fig 3B, column 1 vs 2, p = 0.13). *Dyrk1a* mRNA expression was increased in Δwdr68-9 cells relative to NT1 DM controls (Fig 3B, column 3 vs 2, p<0.05), but not significantly different than NT1 cells in GM (Fig 3B column 3 vs 1). *Dyrk1a* mRNA expression was similar in Δdyrk1a-12 cells and NT1 DM controls (Fig 3B, column 4 vs 2, p = 0.24). These modest shifts in mRNA levels may reflect the action of regulatory feedback loops but do not explain the reductions in DYRK1A protein observed in Δwdr68 cells. Thus, WDR68 was not required for *Dyrk1a* mRNA accumulation suggesting it instead mediates a post-transcriptional step important for DYRK1A protein accumulation or stability.

### WDR68 overexpression induces DYRK1A overexpression

**Fig 4:**
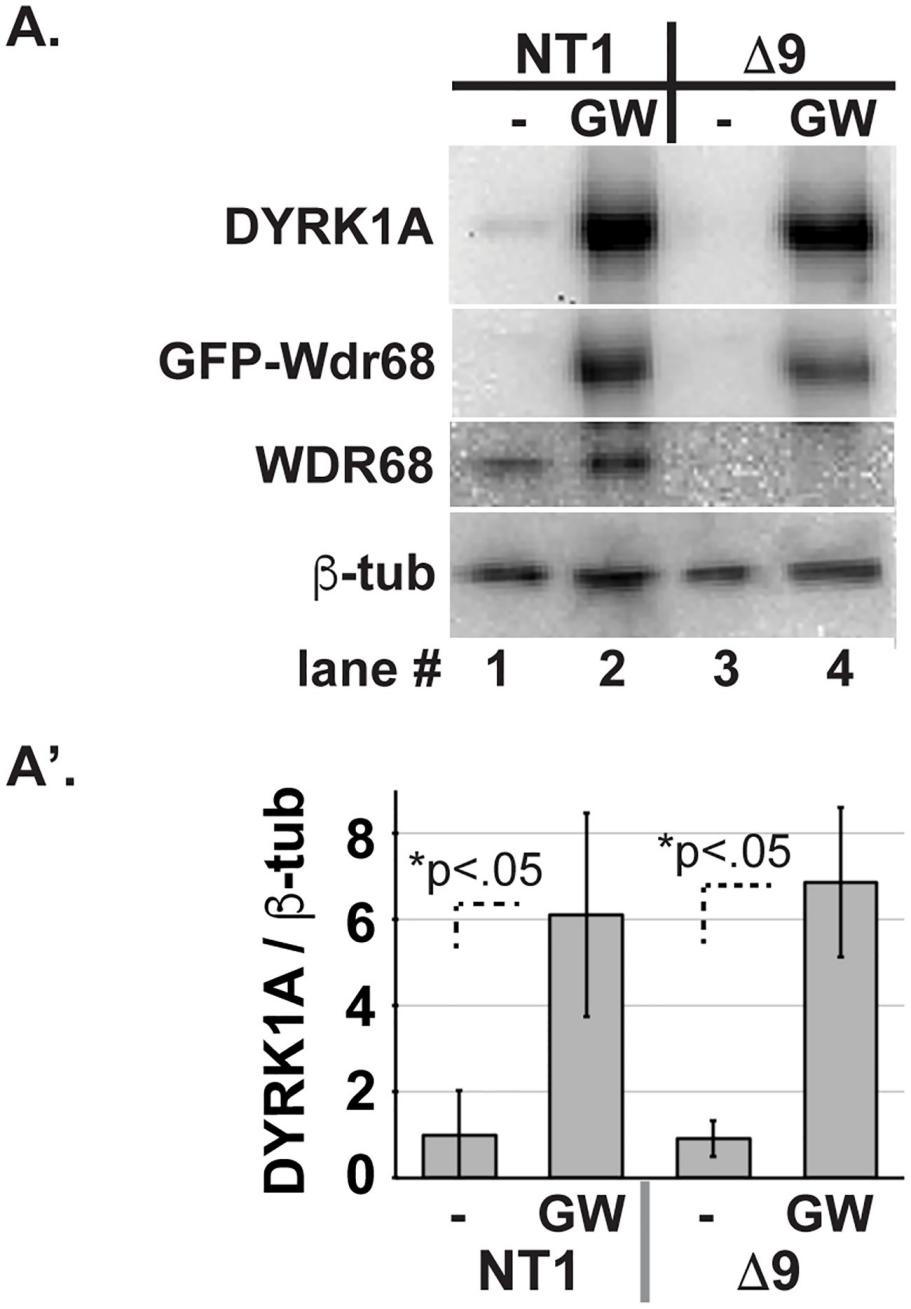
GFP-Wdr68 overexpression increases the level of DYRK1A. A) C2C12 sublines mock (-) or transiently transfected with pEGFP-Wdr68 (GW) in GM. Lanes 1–2 are NT1 control cells. Lanes 3–4 are Δwdr68-9 (Δ9) cells. GFP-Wdr68 panel: Lanes 1 and 3, GFP-Wdr68 fusion protein was absent from mock-transfected cells. Lanes 2 and 4, GFP-Wdr68 fusion protein was detected in transfected cells. DYRK1A panel: Lane 1, endogenous DYRK1A was detected in NT1 cells. Lane 2, endogenous DYRK1A increased in NT1 cells overexpressing GFP-Wdr68. Lane 3, endogenous DYRK1A was not observed in Δwdr68-9 cells. Lane 4, endogenous DYRK1A increased in Δwdr68-9 cells overexpressing GFP-Wdr68. WDR68 panel: Lanes 1 and 2, endogenous WDR68 was detected in NT1 cells. Lanes 3 and 4, endogenous WDR68 expression was not observed in Δwdr68-9 cells. β-tubulin panel: β-tubulin controls indicated similar loading in each lane. A’) Quantitative analysis confirmed significantly increased endogenous DYRK1A levels in GFP-Wdr68 overexpressing NT1 and Δwdr68-9 cells. Three independent biological replicates were analyzed.

Because *Wdr68* loss-of-function reduced DYRK1A level, we next sought to determine whether *Wdr68* gain-of-function would increase DYRK1A level. Therefore, we transiently transfected GFP-Wdr68 into NT1 control and Δwdr68 cells in GM when endogenous DYRK1A levels are low (Fig 4). We readily detected the overexpression of GFP-Wdr68 in NT1 and Δwdr68 cells (Fig 4A, lanes 2 and 4). We likewise readily detected a significant increase in the level of endogenous DYRK1A in the GFP-Wdr68 overexpressing cells (Fig 4A and 4A’, lanes 2 and 4 vs lanes 1 and 3, p<0.05). Endogenous WDR68 was unaffected in NT1 controls (Fig 4A, lanes 1 and 2), and absent from Δwdr68 cells (Fig 4A, lanes 3 and 4), as expected. We examined the levels of β-tubulin as the loading control and for normalization and found them to be similar (Fig 4A and 4A’, lanes 1–4). Thus, WDR68 overexpression increased DYRK1A protein level in both control and Δwdr68 cells, respectively.

To determine whether the converse might also be true, we transfected NT1 and NT2 control cells with GFP-DYRK1A and examined the level of endogenous WDR68 (S1 Fig). We found no effect on the level of endogenous WDR68 or DYRK1B in either the mouse or human cells (S1A Fig). Thus, DYRK1A appears to not regulate the level of WDR68 protein.

### Proteasome inhibition does not alter DYRK1A level

**Fig 5:**
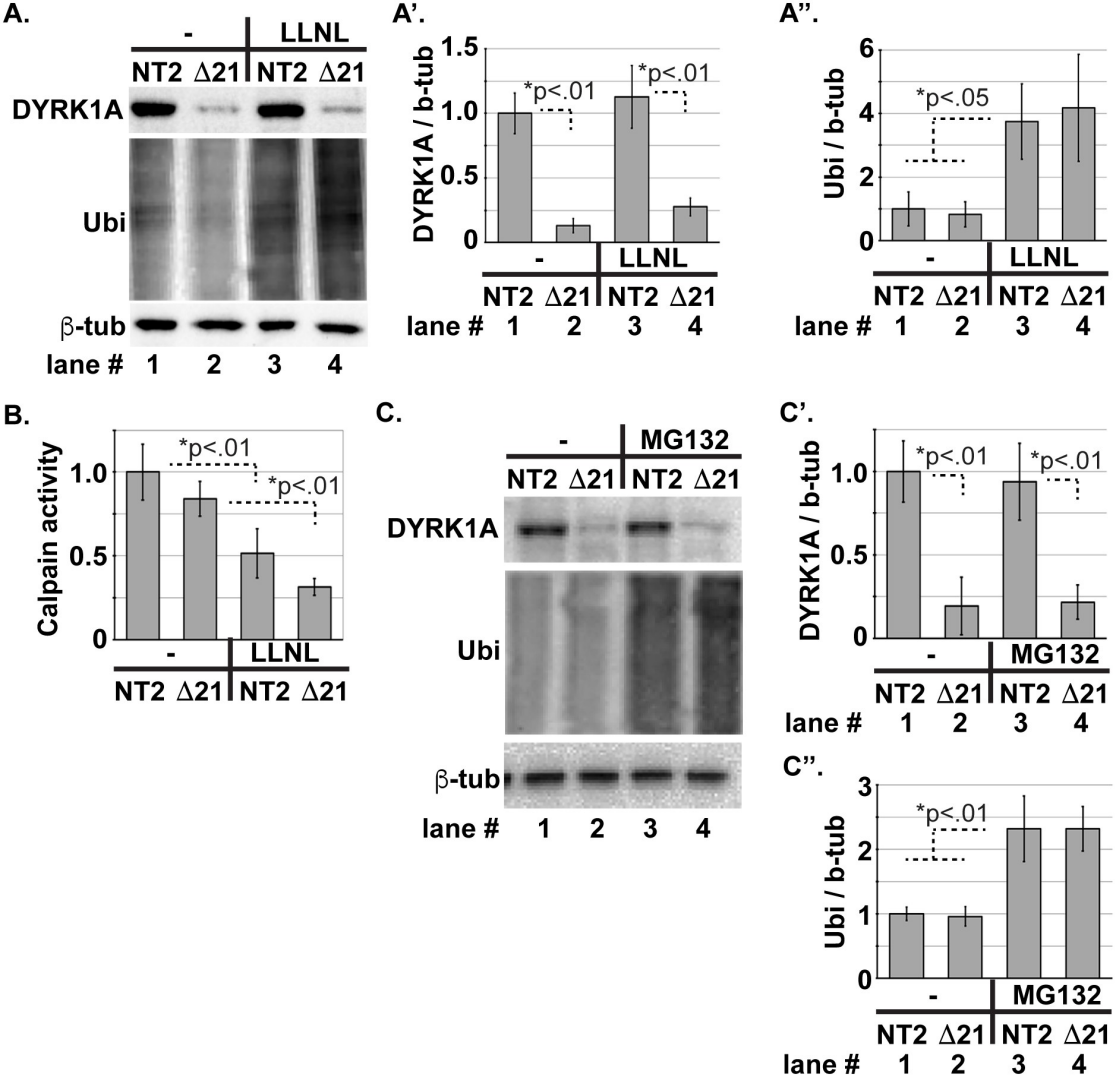
Proteasome inhibitors do not increase DYRK1A level. A) HeLa NT2 and Δwdr68-21 (Δ21) cells mock (-) or treated with 50μM LLNL for 6 hours. DYRK1A panel: Lanes 1 and 3, endogenous DYRK1A was readily detected in NT1 cells and unaffected by exposure to 50μM LLnL. Lanes 2 and 4, endogenous DYRK1A was reduced in Δwdr68-9 cells and unaffected by exposure to 50μM LLnL. Ubiquitin panel: Lanes 1 and 2, low levels of ubiquitinated proteins were readily detected in mock treated cells. Lanes 3 and 4, LLnL induced accumulation of ubiquitinated proteins. β-tubulin panel: β-tubulin controls indicated similar loading in each lane. A’) Quantitative analysis revealed no significant change in endogenous DYRK1A level in response to 6 hours LLnL exposure. A”) Quantitative analysis confirmed significantly increased levels of ubiquitinated proteins in response to 6 hours LLnL exposure. Three independent biological replicates were analyzed. B) Calpain-Glo activity assay on NT2 and Δwdr68-21 cells mock (-) or treated with 50μM LLnL. Arbitrary light units from substrate hydrolysis are expressed per microgram of cell protein extract. Three independent biological replicates were analyzed. C) HeLa NT2 and Δwdr68-21 cells mock (-) or treated with 50μM MG132 for 8 hours. DYRK1A panel: Lanes 1 and 3, endogenous DYRK1A was readily detected in NT2 cells and unaffected by exposure to 50μM MG132. Lanes 2 and 4, endogenous DYRK1A was reduced in Δwdr68-21 cells and unaffected by exposure to 50μM MG132. Ubiquitin panel: Lanes 1 and 2, low levels of ubiquitinated proteins were readily detected in mock treated cells. Lanes 3 and 4, MG132 induced accumulation of ubiquitinated proteins. β-tubulin panel: β-tubulin controls indicated similar loading in each lane. C’) Quantitative analysis revealed no significant change in endogenous DYRK1A level in response to 8 hours MG132 exposure. Three independent biological replicates were analyzed. C”) Quantitative analysis confirmed significantly increased levels of ubiquitinated proteins in response to 8 hours MG132 exposure. Three independent biological replicates were analyzed.

The E3 ligase SCF^**ßTRCP**^ has been reported to mediate ubiquitination and proteasome-dependent degradation of DYRK1A [55]. Likewise, WDR68 has also been implicated in ubiquitination-mediated processes [26, 27]. Therefore, we examined whether the proteasome inhibitors LLnL [56], MG132 [57], or epoxomicin [58] might restore DYRK1A levels in Δwdr68 cells (Fig 5, S2A Fig). First, we treated HeLa NT2 control and Δwdr68 cells with 50μM LLnL for 6 hours. Although we again observed the significantly reduced level of DYRK1A in Δwdr68 cells relative to controls (Fig 5A and 5A’ compare lanes 1 to 2 and 3 to 4, p <0.01), we found that LLnL had no significant effect on the level of DYRK1A in Δwdr68 cells (Fig 5A, compare lanes 2 to 4). As a control for LLnL-mediated proteasome inhibition [59], we examined the expression of total ubiquitinated proteins using an anti-Ubiquitin antibody and found the expected rise in ubiquitinated proteins (Fig 5A and 5A”, compare lanes 1 and 2 to lanes 3 and 4, p<0.05). We examined the expression levels of β-tubulin as the loading control and for normalization and found them to be similar (Fig 5A, 5A’ and 5A”, lanes 1–4). Notably, LLnL is also an inhibitor of CALPAIN1 [60], and DYRK1A is subject to proteolysis by Calpain 1 [61]. Therefore, we examined whether LLnL also inhibited endogenous Calpain proteolytic activity in our cell extracts (Fig 5B). We readily detected Calpain activity in mock-treated NT2 and Δwdr68 cells (Fig 5B, columns 1 and 2), and the amount of activity was significantly lower in LLnL-treated cells (Fig 5B, columns 3 and 4, p<0.01). Next, we treated NT2 control and Δwdr68 cells with 50μM MG132 for 8 hours. Although we again observed the significantly reduced level of DYRK1A in Δwdr68 cells relative to controls (Fig 5C and 5C’ compare lanes 1 to 2 and 3 to 4, p <0.01), we found no effect on the expression level of DYRK1A (Fig 5C and 5C’, compare lanes 2 and 4). As a control for MG132-mediated proteasome inhibition [59], we examined the expression of total ubiquitinated proteins using an anti-Ubiquitin antibody and found the expected rise in ubiquitinated proteins (Fig 5C and 5C”, compare lanes 1 and 2 vs lanes 3 and 4, p<0.01). We examined the expression levels of β-tubulin as the loading control and for normalization and found them to be similar (Fig 5C, 5C’ and 5C”, lanes 1–4). We similarly treated C2C12 NT1 and Δwdr68 cells with 50μM LLnL (S2A Fig) or 50μM epoxomicin (S2B Fig) and found no change in DYRK1A levels.

Because protein destruction can also be regulated via an autophagy mechanism [62, 63], we also examined whether inhibition of lysosomal fusion events might alter DYRK1A levels. We found no effect on the level of DYRK1A in Δwdr68 cells treated with 12.5μM chloroquine (S2C and S2C’ Fig).

## WDR68 is required for cells to reach normal levels of DYRK1B

**Fig 6:**
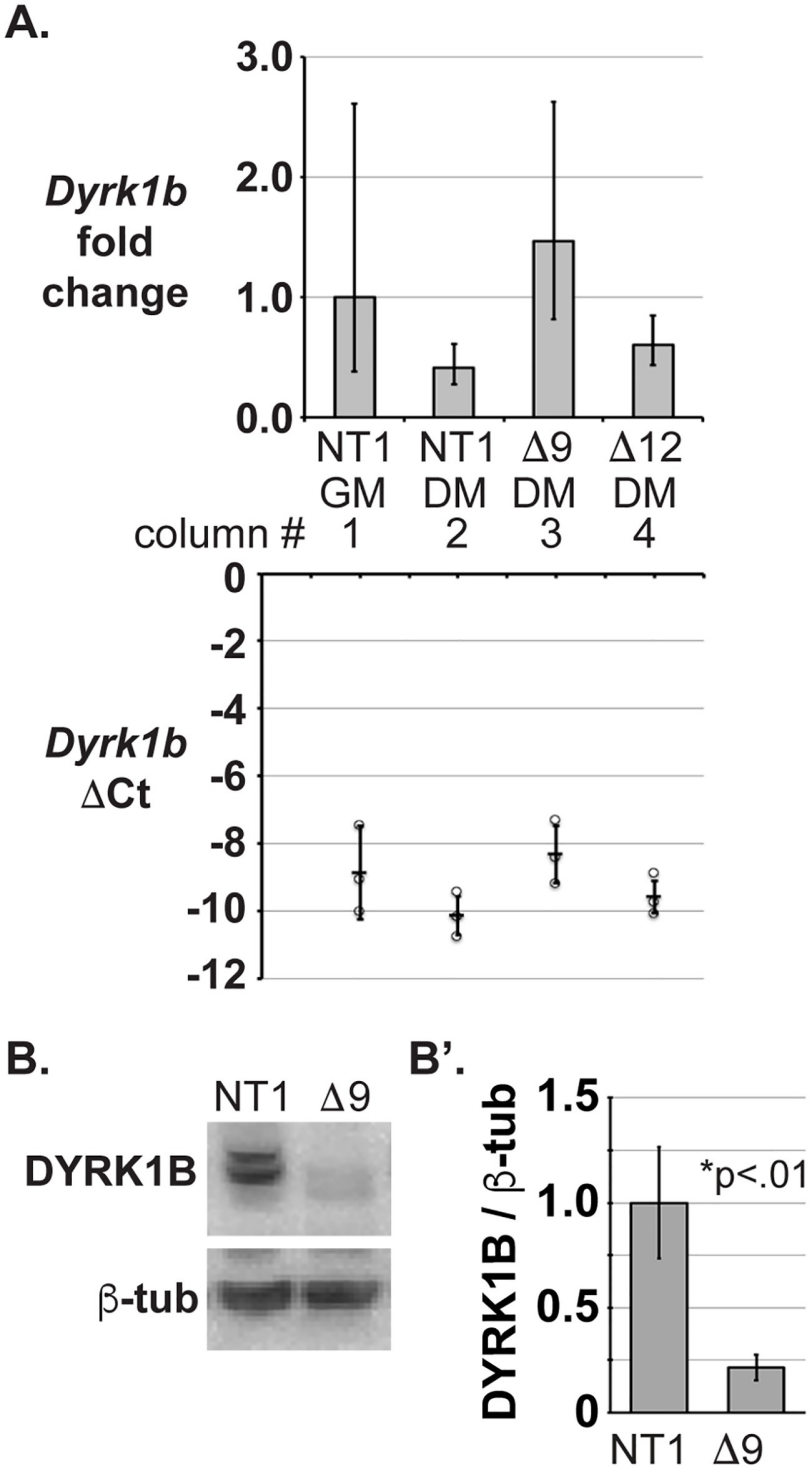
WDR68 is required for normal levels of DYRK1B. A) RT-qPCR analysis of *Dyrk1b* mRNA expression in C2C12 sublines. Column 1 is NT1 cells in GM. Column 2 is NT1 cells in DM. Column 3 is Δwdr68-9 (Δ9) cells in DM. Column 4 is Δdyrk1a-12 (Δ12) cells in DM. The bottom graph shows the individual ΔCt values (circles) of replicates and the average ΔCt (horizontal bar) -/+ standard deviations. The top graph shows the 2^ΔΔCt^ values (fold change relative to NT1 GM) with corresponding low and high ranges. B) Western blot analysis of C2C12 NT1 and Δwdr68-9 cells in DM. DYRK1B panel: Lane 1, DYRK1B was readily detected in NT1 cells. Lane 2, DYRK1B expression was reduced in Δwdr68-9 cells. β-tubulin panel: β-tubulin controls indicated similar loading in each lane. B’) Quantitative analysis confirmed significantly reduced DYRK1B expression in the Δwdr68 subline. Three independent biological replicates were analyzed.

To determine whether *Wdr68* is required for *Dyrk1b* mRNA accumulation, we performed RT-qPCR analysis on NT1 control, Δwdr68, and Δdyrk1a cells. *Dyrk1b* mRNA was readily and similarly detected in all the samples (Fig 6A, columns 1–4). Next, we examined the levels of DYRK1B protein and found that it was significantly reduced in Δwdr68 cells (Fig 6B and 6B’, compare lanes 1 and 2, p<0.01). We examined the levels of β-tubulin as the loading control and for normalization and found them to be similar (Fig 6B and 6B’, lanes 1–2). Thus, WDR68 is also required for cells to reach normal levels of DYRK1B.

### DYRK1A and DYRK1B are required for the transition from growth to differentiation

**Fig 7:**
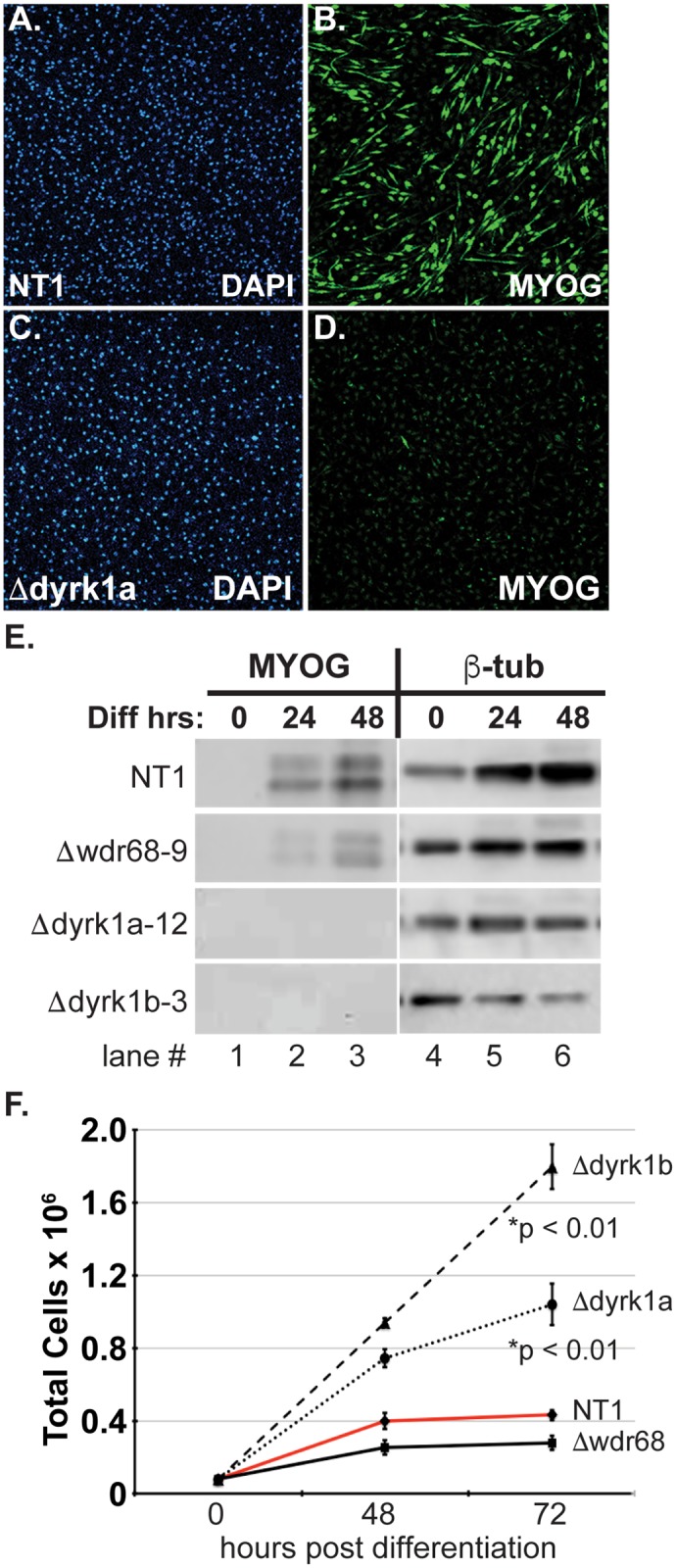
DYRK1A and DYRK1B are required for myogenic differentiation and growth arrest in C2C12 cells. A-D) Confocal fluorescence imaging of C2C12 cells in DM for 48 hours. A) DAPI staining of NT1 cell nuclei. B) Immunofluorescence detection of robust Myogenin (MYOG) induction in differentiating NT1 control cells. C) DAPI staining of Δdyrk1a-12 cell nuclei. D) absence of MYOG induction in Δdyrk1a-12 cells. E) Western blot analysis of MYOG and β-tubulin protein expression. NT1 panel: Lane 1, absence of MYOG in non-differentiating cells. Lanes 2 and 3, induction of MYOG in NT1 control cells at 24hrs and 48hrs post differentiation. Δwdr68-9 panel: Lane 1, absence of MYOG induction in non-differentiating cells. Lanes 2 and 3, impaired accumulation of MYOG in Δwdr68-9 cells at 24hrs and 48hrs post-differentiation. Δdyrk1a-12 panel: Lane 1, absence of MYOG induction in non-differentiating cells. Lanes 2 and 3, Δdyrk1a-12 cells lack MYOG at 24hrs and 48hrs post-differentiation. Δdyrk1b-3 panel: Lane 1, absence of MYOG induction in non-differentiating cells. Lanes 2 and 3, Δdyrk1b-3 cells lack MYOG at 24hrs and 48hrs post-differentiation. β-tubulin panels indicated similar loading in each lane. F) Growth of NT1, Δwdr68-9, Δdyrk1a-12, and Δdyrk1b-3 cell lines over time after the switch to DM. NT1 controls and Δwdr68-9 cells arrested growth by 48hrs post-differentiation. Δdyrk1a-12 and Δdyrk1b-3 cell growth was not stopped by the switch to DM.

Curiously, DM-induction of WDR68 was less robust in Δdyrk1a cells (Fig 1B and 1B”, compare lanes 5 and 6 to lane 4). To determine whether this might reflect a defect in the ability of Δdyrk1a cells to undergo myogenic differentiation, we examined the levels of Myogenin (MYOG) in cells at various times post-differentiation (Fig 7A–7E). The NT1 control cells clearly induced MYOG by 48 hours post-differentiation (Fig 7B and 7E NT1 panel, compare lane 3 to lane 1). In contrast, MYOG was not detected in Δdyrk1a cells (Fig 7D and 7E panel Δdyrk1a-12, compare lane 3 to lane 1). Consistent with previous reports [25], induction of MYOG was less robust in the Δwdr68 cells (Fig 7E panel Δwdr68-9, compare lane 3 to lane 1). For appropriate comparisons, we used CRISPR/Cas9 to derive Δdyrk1b sublines (S3 Fig). Consistent with prior reports [25, 64], MYOG was not detected in the Δdyrk1b cells (Fig 7E, panel Δdyrk1b-3, compare lane 3 to lane 1). We examined the levels of β-tubulin as the loading control and found them to be similar (Fig 7E, β-tub panels, lanes 4–6).

We next compared the growth of the NT1 control cells to that of the Δwdr68, Δdyrk1a, and Δdyrk1b sublines after the induction of differentiation (Fig 7F). As expected based on the readily detected levels of MYOG, the NT1 control and Δwdr68 sublines ceased growth by 48 hours post-differentiation (Fig 7F, NT1 solid red line, Δwdr68 solid black line). The still largely effective growth arrest observed in Δwdr68 cells is likely due to the residual levels of DYRK1A and DYRK1B detected in those cells still mediating this function (Fig 1A and 1A” lane 4; 6B 6B’ lane 2). In contrast, the Δdyrk1a and Δdyrk1b cells failed to arrest growth (Fig 7F, Δdyrk1a dotted line, Δdyrk1b dashed line). We attempted to rescue the MYOG defect by treating cells with the cell cycle inhibitor roscovitine (S4 Fig). However, all concentrations that effectively blocked cell growth also blocked MYOG induction in the NT1 control cells.

## Discussion

### WDR68 is required for normal levels of DYRK1A and DYRK1B

We found that WDR68 is required for normal levels of DYRK1A in both mouse and human cells (Fig 1A and 2A), that partial reduction of WDR68 yielded a partial reduction of DYRK1A level (Fig 1C), and that WDR68 overexpression induces DYRK1A protein level (Fig 4). We similarly found that WDR68 is required for normal levels of DYRK1B (Fig 6). Conversely, we found that DYRK1A is not required for normal levels of WDR68 (Fig 1B), nor did DYRK1A overexpression impact WDR68 levels (S1 Fig). Taken together, these findings support a unidirectional and positive regulatory relationship between WDR68 levels and DYRK1A and DYRK1B levels.

### Mechanisms regulating WDR68, DYRK1A, and DYRK1B

Analysis of *Wdr68* mRNA levels by RT-qPCR revealed similar transcript levels in NT1 cells in GM and DM (Fig 3). This finding was surprising because the level of WDR68 protein, relative to ß-tubulin, actually increased in DM. Likewise, *Dyrk1a* mRNA levels were not induced by the switch from GM to DM but instead appeared to decline slightly. In contrast, DYRK1A protein levels actually increased upon differentiation. Analysis of *Dyrk1a* and *Dyrk1b* transcript levels in Δwdr68 cells did not implicate WDR68 in either transcription-level or mRNA stability-level control of their expression (Figs 3 and 6). These findings indicate that the increase of WDR68, DYRK1A, and DYRK1B proteins observed upon switching cells from GM to DM must be a largely translational or post-translational regulatory event rather than a simple induction of mRNA expression. The factors responsible for these effects on Wdr68 and Dyrk1a remain to be identified.

It is attractive to speculate that WDR68 binding to DYRK1A and DYRK1B protects the kinases from destruction via a steric occlusion mechanism or the recruitment of another stabilizing factor. The adaptor role of many WD40 repeat domain proteins is consistent with this possibility [20]. For example, Glenewinkel et al., found that WDR68 binding to E1A enhances DYRK1A-mediated phosphorylation of E1A likely via forced proximity of the kinase to the substrate. This close proximity also masks the nuclear localization signals such that the entire E1A-WDR68-DYRK1A complex redistributes to the cytoplasm instead of the nucleus. Thus WDR68 binding to DYRK1A may similarly recruit or occlude a factor that stabilizes or degrades DYRK1A, respectively. Nonetheless, the identity of such a factor remains unclear.

Because DYRK1A and WDR68 have both been implicated in ubiquitin-proteasome processes [26, 27, 55], we examined three different proteasome inhibitors and one autophagy inhibitor to see if WDR68 might protect DYRK1A from ubiquitin-dependent proteasomal or lysosomal destruction. However, none of the inhibitors were able to restore DYRK1A levels in Δwdr68 cells (Fig 5, S2 Fig). Thus, the mechanism by which WDR68 regulates DYRK1A and DYRK1B levels remains elusive. One possibility is that WDR68 protects DYRK1A and DYRK1B from a currently unidentified DYRK1-specific protease. Notably, DYRK1A is known to be a proteolytic substrate of Calpain 1 [61]. The proteasome inhibitor LLnL is known by many synonyms including MG101 and Calpain Inhibitor I. Nonetheless, it failed to significantly restore DYRK1A levels (Fig 5A) even though it appeared to be effective as a Calpain inhibitor (Fig 5B). While target-specific cleavage events often yield semi-stable fragments of the targeted protein, detection of such fragments can be challenging and antibody-dependent.

An interesting feature of the DYRK1 kinases is the strict requirement of an intramolecular autophosphorylation event in order to yield mature kinase capable of phosphorylating other substrates [30, 65, 66]. While it is clear that this is an intrinsic and possibly co-translational activity of mammalian DYRK1 [66, 67], certain disease causing mutations in DYRK1B are more dependent on chaperone activity for kinase maturation and yield high levels of insoluble kinase [68]. Perhaps WDR68 binding facilitates some aspect of kinase maturation and the reduced levels of kinase we report here are actually loss of the soluble kinases to a highly insoluble form. Alternatively, WDR68 may somehow enhance *DYRK1* mRNA translation, perhaps independent from its physical interaction with DYRK1 proteins.

### A potential DYRK1A-WDR68 co-activator complex

WDR68 facilitates DYRK1A-mediated co-activation of reporter activity (Fig 3). This finding is easily explained by the aforementioned reductions of DYRK1A levels in cells lacking WDR68 (Figs 1 and 2). The remaining level of reporter activity may reflect the presence of other kinases partially redundant with DYRK1A, such as DYRK1B [38, 41]. Alternatively, it may reflect the instrinsic activity associated with the transcription factor(s) that directly bind the TCTCGCGAGA palindrome [41]. This sequence is among the most enriched and conserved elements in human promoters [69, 70]. Notably, the DNA-binding transcription factor KAISO, that recruits the transcriptional co-repressor SMRT, tightly binds only the CpG-methylated form of this palindrome [71]. However, multiple large-scale protein-protein interaction screens that did detect a DYRK1A-WDR68 interaction did not find either a DYRK1A-KAISO or a WDR68-KAISO interaction [72–74]. Thus, an intriguing possibility is that an as yet unidentified transcription factor tethers the DYRK1A-WDR68 co-activator complex to the unmethylated TCTCGCGAGA palindrome to regulate proliferation/cell-cycle genes. This could complement the role DYRK1A plays in DREAM complex assembly [38, 39], and together the loss of these activities may underlie the loss of differentiation and growth arrest we observed in Δdyrk1a and Δdyrk1b C2C12 cells (Fig 7). While the Δdyrk1a and Δdyrk1b cells failed to arrest growth, the Δwdr68 cells arrested growth normally (Fig 7F). At first this seems at odds to the finding that DYRK1A and DYRK1B levels are reduced in Δwdr68 cells, but it is important to note that residual levels of DYRK1A and DYRK1B are present in Δwdr68 cells. The residual levels of DYRK1A in Δwdr68 cells likely also contribute to the remaining DYRK1A-reporter activity. Thus, the reductions of DYRK1A and DYRK1B levels in Δwdr68 cells, while significant, are insufficient to impair cell cycle arrest.

### Future directions for DS and craniofacial research

DS patients develop facial dysmorphology suggesting a role for DYRK1A in craniofacial development [75]. WDR68 is important for craniofacial development [3], and tightly binds DYRK1A [15]. DYRK1A is an attractive target for inhibitor-based therapies and enthusiasm is growing for this approach to treating various aspects of DS [76, 77]. Our findings here complement earlier findings [19], to similarly suggest WDR68 as a potential additional target for DS treatment to modulate the levels of DYRK1A. However, the lack of knowledge on whether WDR68 plays any role in DS pathology and how DYRK1 proteins function in craniofacial development are significant limitations. Additional work in these areas will be needed.

## Supporting information

S1 Table CRISPR/Cas9-mediated C2C12 cell deletion subline alleles. (DOCX)

S2 Table CRISPR/Cas9-mediated HeLa cell deletion subline alleles. (DOCX)

S1 Fig GFP-DYRK1A overexpression does not increase WDR68 levels. A) Western blot analysis of C2C12 NT1 cells. GFP-DYRK1A and endogenous DYRK1A panel: Lane 1, GFP-DYRK1A fusion was absent and endogenous DYRK1A was readily detected. Lane 2, transfected GFP-DYRK1A and endogenous DYRK1A were readily detected. DYRK1B panel: Lane 1 and 2, endogenous DYRK1B was readily detected and not altered by GFP-DYRK1A overexpression. WDR68 panel: Lane 1 and 2, endogenous WDR68 was readily detected and not increased by GFP-DYRK1A overexpression. GFP panel: Lane 1, transfected GFP was readily detected. Lane 2, GFP was absent. β-tubulin panel: β-tubulin controls indicated similar loading in each lane. B) Western blot analysis of HeLa NT2 cells. GFP-DYRK1A and endogenous DYRK1A panel: Lane 1, GFP-DYRK1A fusion was absent and endogenous DYRK1A was readily detected. Lane 2, transfected GFP-DYRK1A and endogenous DYRK1A were readily detected. DYRK1B panel: Lane 1 and 2, endogenous DYRK1B was readily detected and not altered by GFP-DYRK1A overexpression. WDR68 panel: Lane 1 and 2, endogenous WDR68 was readily detected and not increased by GFP-DYRK1A overexpression. GFP panel: Lane 1, transfected GFP was readily detected. Lane 2, GFP was absent. β-tubulin panel: β-tubulin controls indicated similar loading in each lane.
(TIF)

S2 Fig Chloroquine does not increase DYRK1A levels. Western blot analysis of HeLa NT2 and Δwdr68-21 cells. B) NT2 and Δwdr68-21 cells mock (-) or treated with 50μM epoxomicin for 8 hours. DYRK1A panel: Lanes 1 and 3, endogenous DYRK1A was readily detected in NT1 cells and unaffected by exposure to 50μM epoxomicin. β-tubulin panel: β-tubulin controls indicated similar loading in each lane. A) HeLa NT2 and Δwdr68-21 cells in vehicle DMSO (-) or treated with 12.5μM CQ for 8 hours. DYRK1A panel: Lanes 1 and 3, endogenous DYRK1A was readily detected in NT1 cells and unaffected by exposure to 12.5μM CQ. Lanes 2 and 4, endogenous DYRK1A expression was reduced in Δwdr68-21 cells and unaffected by exposure to 12.5μM CQ. β-tubulin panel: β-tubulin controls indicated similar loading in each lane. A’) Quantitative analysis revealed no significant change in endogenous DYRK1A expression in response to 8 hours CQ exposure.
(TIF)

S3 Fig Reduced DYRK1B levels in Δdyrk1b C2C12 sublines. Western blot analysis of C2C12 NT1 and Δdyrk1b cells. A) DYRK1B panel: Lane 1, DYRK1B was readily detected in NT1 cells. Lanes 2–4, reduced DYRK1B expression in Δdyrk1b-3, -4, and -7 cells. β-tubulin panel: β-tubulin controls indicated similar loading in each lane. A’) Quantitative analysis confirmed significantly reduced DYRK1B expression in the Δdyrk1b sublines.
(TIF)

S4 Fig Cell cycle inhibition does not restore myogenic differentiation in Δwdr68, Δdyrk1a, orΔdyrk1b C2C12 cells. Western blot analysis on various sublines at 24 hours post-differentiation. A) MYOG panel: Lanes 1–4, MYOG was detected in NT1 control cells but not in Δwdr68-9, Δdyrk1a-12 or Δdyrk1b-3. Lanes 5–8, roscovitine treatment for 24 hours at the indicated concentrations did not restore MYOG levels. β-tubulin panel: β-tubulin controls indicated similar loading in each lane.
(TIF)

S1 Appendix Uncropped western blots for all figures. (PDF)

S2 Appendix Quantifications. (XLSX)
