# Supplementary material for: DCAF7/WDR68 is required for normal levels of DYRK1A and DYRK1B
Source: PLoS One. 2018 Nov 29;13(11):e0207779. doi: 10.1371/journal.pone.0207779 (PMC6264848; doi:10.1371/journal.pone.0207779)
Supplement: S1 Table — (DOCX) [file pone.0207779.s001.docx]

**S1 Table. CRISPR/Cas9-mediated C2C12 cell deletion subline alleles.**

| **NAME** | **SEQUENCE** | **INDEL** |
| --- | --- | --- |
| *Dyrk1a* wildtype | TGTGTAACCCC**AAA/CGGAGTGCAATCAAGAT**TGTT |  |
| dyrk1a-∆2a | TGTGTAA------- ----------TCAAGATTGTT | -17 |
| dyrk1a-∆2b | TGTGTAACCCCAAAgCGGAGTGCAATCAAGATTGTT | +1 |
| **SAMPLE** | **SEQUENCE** | **INDEL** |
| *Dyrk1a* wildtype | GTCTCTTTGAACCT**AAC/ACGAAAGTTTGCGCAAC**AGATGTG |  |
| dyrk1a-∆12a | GTCTCTTTGAAC----- -----------------AGATGTG | -22 |
| dyrk1a-∆12b | GTCTCTTTGAACCTAAC ----AAGTTTGCGCAACAGATGTG | -4 |
| **SAMPLE** | **SEQUENCE** | **INDEL** |
| *Dyrk1b* wildtype | TGTCCTACAACCT**GTA/CGACCTCCTCCGCAACA**CACACTTT |  |
| dyrk1b-∆3a | TGTCCTACAACCT--- ------CCTCCGCAACACACACTTT | -9 |
| dyrk1b-∆3b | TGTCCTACAACCT--- ---------CCGCAACACACACTTT | -12 |
| dyrk1b-∆4a | TGTCCTACAACCT--- ------CCTCCGCAACACACACTTT | -9 |
| dyrk1b-∆4b | left arm -437bp, right arm -143bp | -580 |
| dyrk1b-∆7a | TGTCCTACAACCTGTA ------CCTCCGCAACACACACTTT | -6 |
| dyrk1b-∆7b | TGTCCTACAACCTGTAaCGACCTCCTCCGCAACACACACTTT | +1 |

Bold upper case indicates gRNA target sequence in wildtype, “/” indicates predicted Cas9 cleavage site, “-“ indicates deleted bases, lower case indicates inserted bases.
