# Supplementary material for: DCAF7/WDR68 is required for normal levels of DYRK1A and DYRK1B
Source: PLoS One. 2018 Nov 29;13(11):e0207779. doi: 10.1371/journal.pone.0207779 (PMC6264848; doi:10.1371/journal.pone.0207779)
Supplement: S2 Table — (DOCX) [file pone.0207779.s002.docx]

**S2 Table. CRISPR/Cas9-mediated HeLa cell deletion subline alleles.**

| **SAMPLE** | **SEQUENCE** | **INDEL** |
| --- | --- | --- |
| *WDR68* wildtype | TGGCAACAA**GCGGTGACTATCTCCGT/GTG**TGGAGG |  |
| wdr68-∆3a | TGGCAACAAGCGGTGACTATCTCCGTtGTGTGGAGG | +1 |
| wdr68-∆3b | TGGCAACAAGCGGTGA----------gactgagcaagagcagacggctggaaaatctgatcgcccagctgcccggcgagaagaagaatggcctgttcggaaacctgattgccctgagcctgggcctgacccccaacttcaagagcaacttcgacctggccgaggatgccaaactgcagctgagcaaggacacctacgacgacgacctggacaacctgctggcccagatcggcgaccagtacgccgacctgtttctggccgccaagaacctgtccgacgccatcctgctgagcgacatcctgagagtgaacaccgagatcaccaaggcat GTGTGGAGG | -10 +303 |
| **SAMPLE** | **SEQUENCE** | **INDEL** |
| *WDR68* wildtype | GCAGAAACACCT**TTG/ACCACCCATACCCCACC**ACAAAGCTCATGT |  |
| wdr68-∆21a | GCAGAAACACCTTTGaACCACCCATACCCCACCACAAAGCTCATGT | +1 |
| wdr68-∆21b | GCAGAAACACCTTTG -aCACCCATACCCCACCACAAAGCTCATGT | -2 +1 |
| wdr68-∆24a | GCAGAAACACCTTTGaaaCACCCATACCCCACCACAAAGCTCATGT | -2 +3 |
| wdr68-∆24b | GCAGAAACACC---- ------CATACCCCACCACAAAGCTCATGT | -10 |

Bold upper case indicates gRNA target sequence in wildtype, “/” indicates predicted Cas9 cleavage site, “-“ indicates deleted bases, lower case indicates inserted bases.
