# Supplementary figures and images for: DCAF7/WDR68 is required for normal levels of DYRK1A and DYRK1B
Source: PLoS One. 2018 Nov 29;13(11):e0207779. doi: 10.1371/journal.pone.0207779 (PMC6264848; doi:10.1371/journal.pone.0207779)

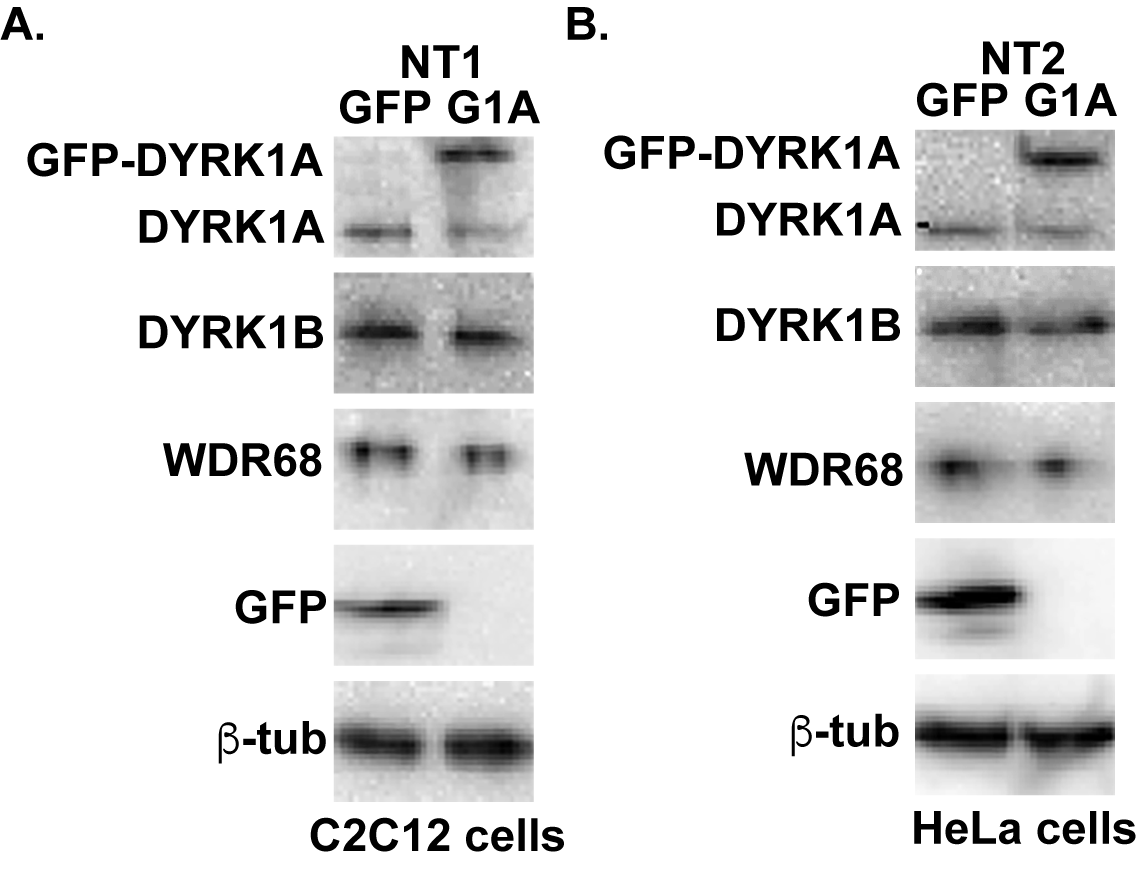

Supplement: S1 Fig — A) Western blot analysis of C2C12 NT1 cells. GFP-DYRK1A and endogenous DYRK1A panel: Lane 1, GFP-DYRK1A fusion was absent and endogenous DYRK1A was readily detected. Lane 2, transfected GFP-DYRK1A and endogenous DYRK1A were readily detected. DYRK1B panel: Lane 1 and 2, endogenous DYRK1B was readily detected and not altered by GFP-DYRK1A overexpression. WDR68 panel: Lane 1 and 2, endogenous WDR68 was readily detected and not increased by GFP-DYRK1A overexpression. GFP panel: Lane 1, transfected GFP was readily detected. Lane 2, GFP was absent. β-tubulin panel: β-tubulin controls indicated similar loading in each lane. B) Western blot analysis of HeLa NT2 cells. GFP-DYRK1A and endogenous DYRK1A panel: Lane 1, GFP-DYRK1A fusion was absent and endogenous DYRK1A was readily detected. Lane 2, transfected GFP-DYRK1A and endogenous DYRK1A were readily detected. DYRK1B panel: Lane 1 and 2, endogenous DYRK1B was readily detected and not altered by GFP-DYRK1A overexpression. WDR68 panel: Lane 1 and 2, endogenous WDR68 was readily detected and not increased by GFP-DYRK1A overexpression. GFP panel: Lane 1, transfected GFP was readily detected. Lane 2, GFP was absent. β-tubulin panel: β-tubulin controls indicated similar loading in each lane. (TIF) [file pone.0207779.s003.tif]

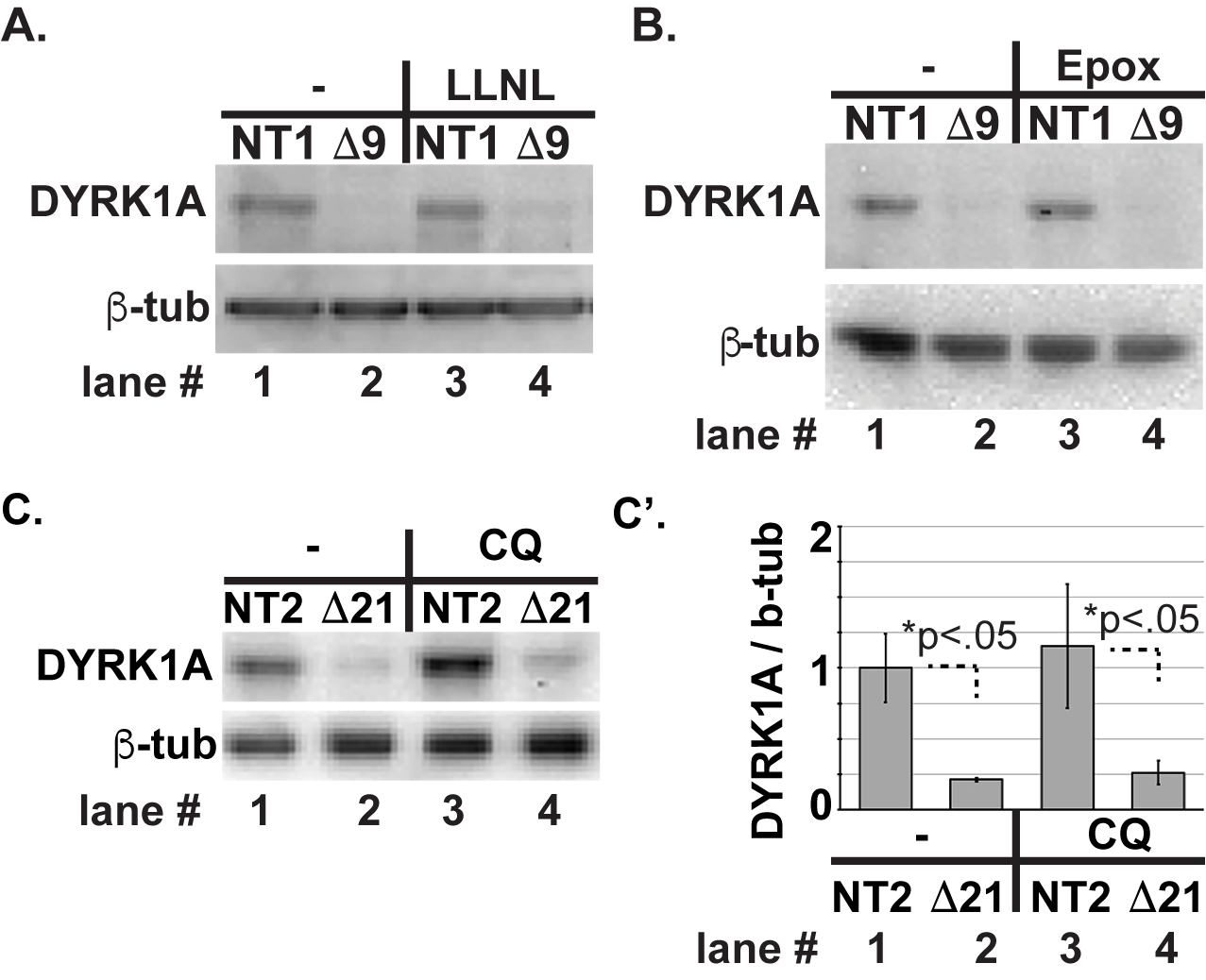

Supplement: S2 Fig — Western blot analysis of HeLa NT2 and Δwdr68-21 cells. B) NT2 and Δwdr68-21 cells mock (-) or treated with 50μM epoxomicin for 8 hours. DYRK1A panel: Lanes 1 and 3, endogenous DYRK1A was readily detected in NT1 cells and unaffected by exposure to 50μM epoxomicin. β-tubulin panel: β-tubulin controls indicated similar loading in each lane. A) HeLa NT2 and Δwdr68-21 cells in vehicle DMSO (-) or treated with 12.5μM CQ for 8 hours. DYRK1A panel: Lanes 1 and 3, endogenous DYRK1A was readily detected in NT1 cells and unaffected by exposure to 12.5μM CQ. Lanes 2 and 4, endogenous DYRK1A expression was reduced in Δwdr68-21 cells and unaffected by exposure to 12.5μM CQ. β-tubulin panel: β-tubulin controls indicated similar loading in each lane. A’) Quantitative analysis revealed no significant change in endogenous DYRK1A expression in response to 8 hours CQ exposure. (TIF) [file pone.0207779.s004.tif]

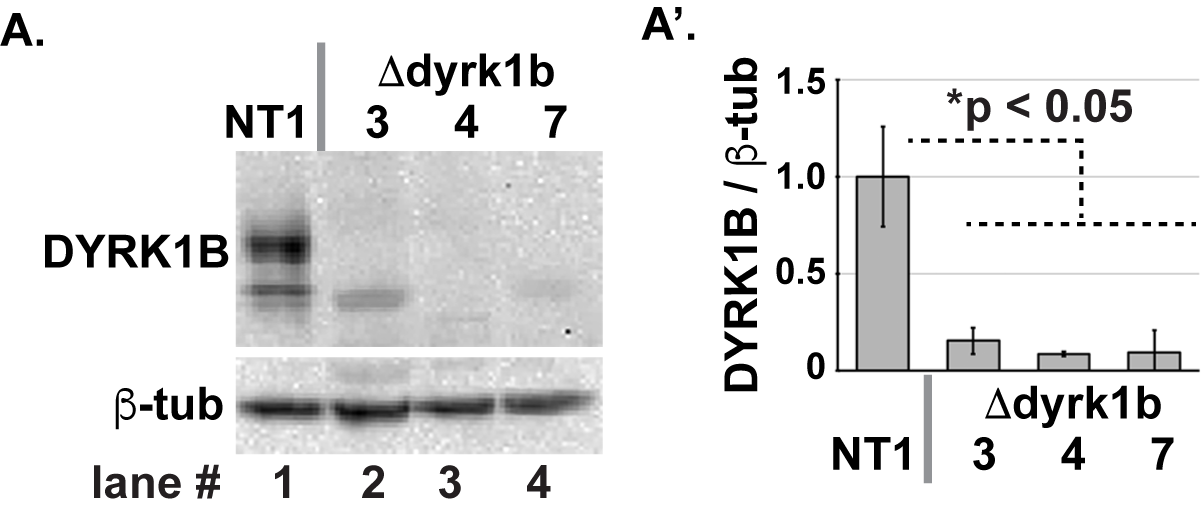

Supplement: S3 Fig — Western blot analysis of C2C12 NT1 and Δdyrk1b cells. A) DYRK1B panel: Lane 1, DYRK1B was readily detected in NT1 cells. Lanes 2–4, reduced DYRK1B expression in Δdyrk1b-3, -4, and -7 cells. β-tubulin panel: β-tubulin controls indicated similar loading in each lane. A’) Quantitative analysis confirmed significantly reduced DYRK1B expression in the Δdyrk1b sublines. (TIF) [file pone.0207779.s005.tif]

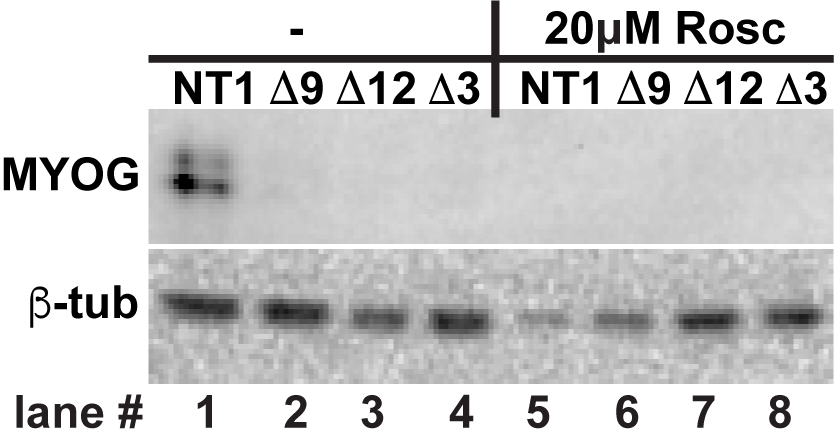

Supplement: S4 Fig — Western blot analysis on various sublines at 24 hours post-differentiation. A) MYOG panel: Lanes 1–4, MYOG was detected in NT1 control cells but not in Δwdr68-9, Δdyrk1a-12 or Δdyrk1b-3. Lanes 5–8, roscovitine treatment for 24 hours at the indicated concentrations did not restore MYOG levels. β-tubulin panel: β-tubulin controls indicated similar loading in each lane. (TIF) [file pone.0207779.s006.tif]
