# Supplementary material for: DCAF7/WDR68 is required for normal levels of DYRK1A and DYRK1B
Source: PLoS One. 2018 Nov 29;13(11):e0207779. doi: 10.1371/journal.pone.0207779 (PMC6264848; doi:10.1371/journal.pone.0207779)
Supplement: S1 Appendix — (PDF) [file pone.0207779.s007.pdf]

Figure 1A

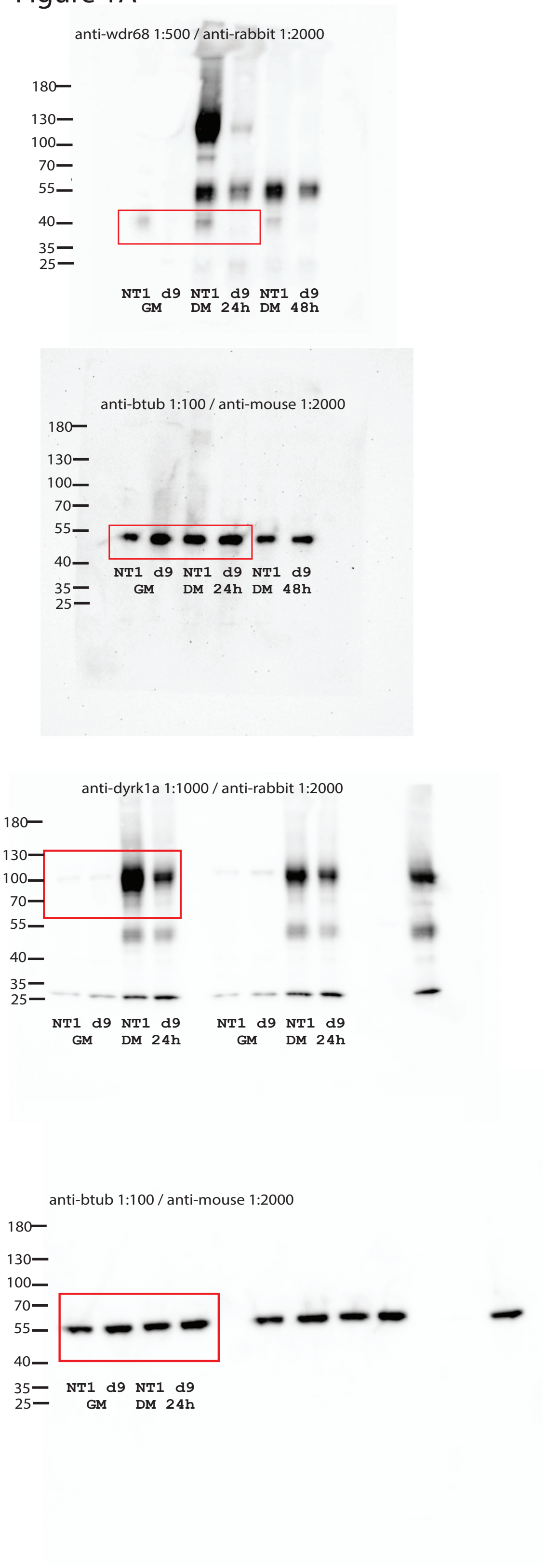

Figure 1B

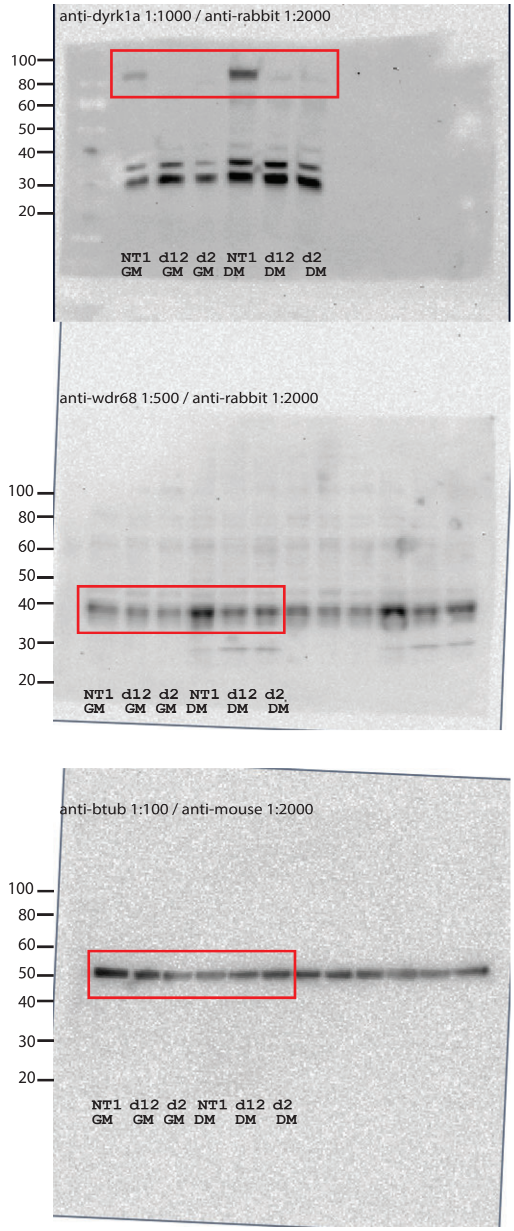

Figure 1C

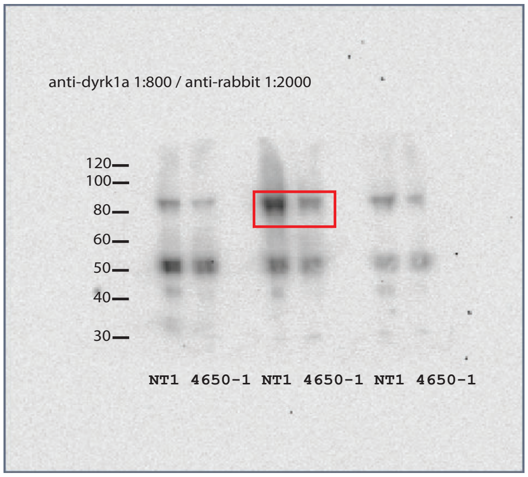

anti-dyrk1a 1:800 / anti-rabbit 1:2000
120
100
80
60
50
40
30
NT1 4650-1 NT1 4650-1 NT1 4650-1

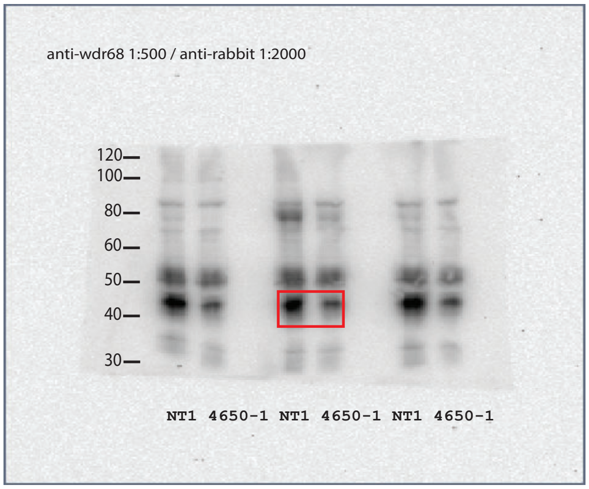

anti-wdr68 1:500 / anti-rabbit 1:2000
120
100
80
60
50
40
30
NT1 4650-1 NT1 4650-1 NT1 4650-1

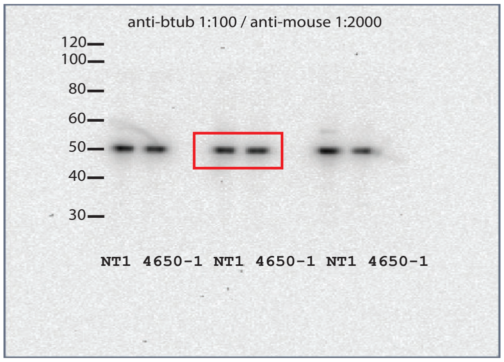

anti-btub 1:100 / anti-mouse 1:2000
120
100
80
60
50
40
30
NT1 4650-1 NT1 4650-1 NT1 4650-1

Figure 2A left

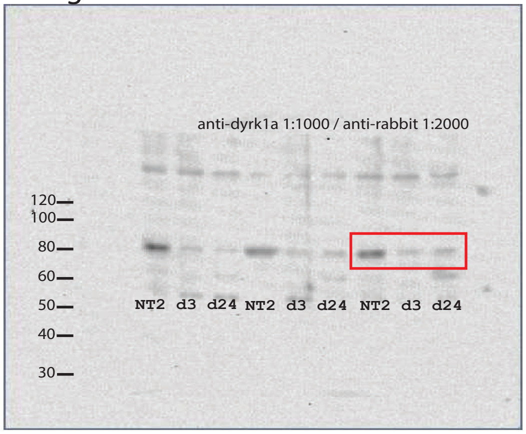

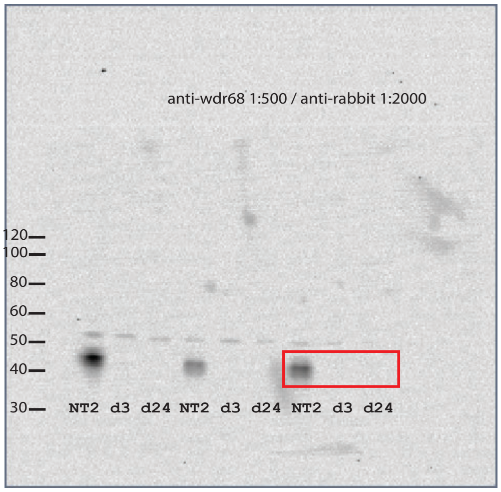

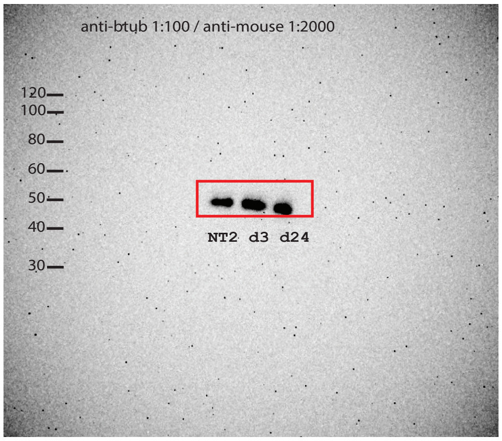

Figure 2A right

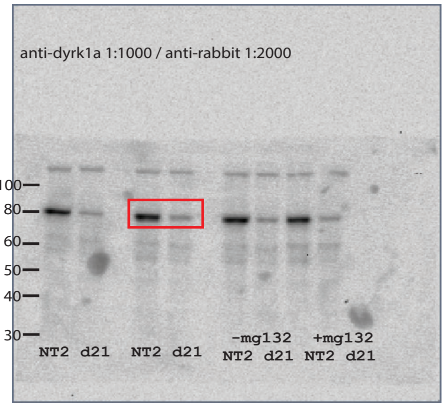

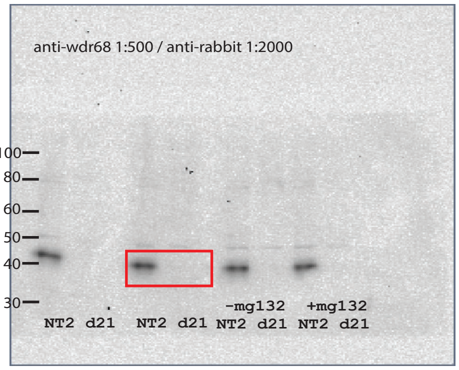

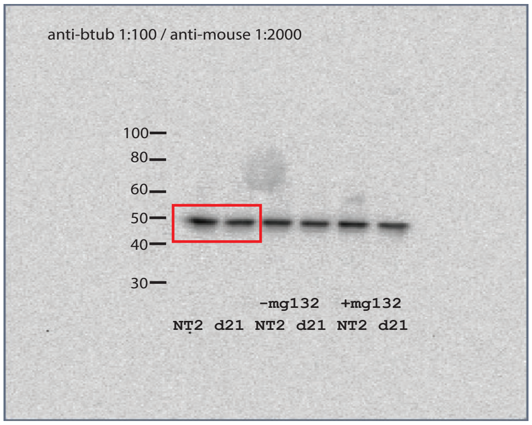

Figure 4A

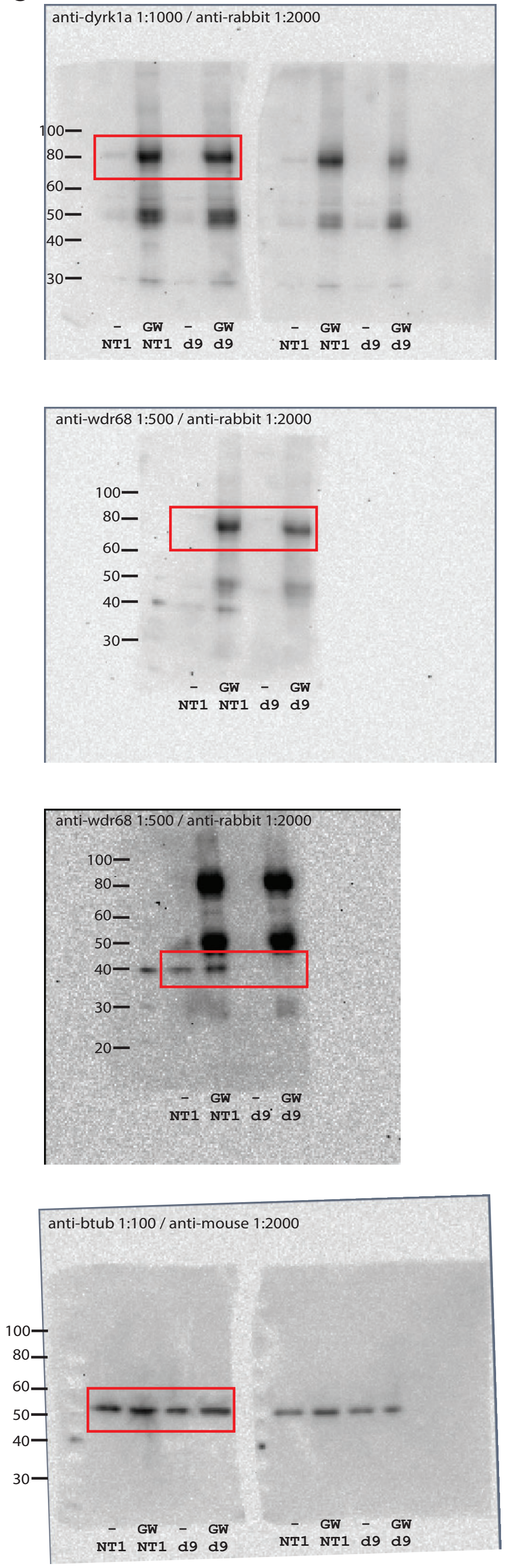
anti-dyrk1a 1:1000 / anti-rabbit 1:2000
100
80
60
50
40
30
- GW - GW - GW - GW
NT1 NT1 d9 d9 NT1 NT1 d9 d9
anti-wdr68 1:500 / anti-rabbit 1:2000
100
80
60
50
40
30
- GW - GW
NT1 NT1 d9 d9
anti-wdr68 1:500 / anti-rabbit 1:2000
100
80
60
50
40
30
20
- GW - GW
NT1 NT1 d9 d9
anti-btub 1:100 / anti-mouse 1:2000
100
80
60
50
40
30
- GW - GW - GW - GW
NT1 NT1 d9 d9 NT1 NT1 d9 d9

Figure 5A

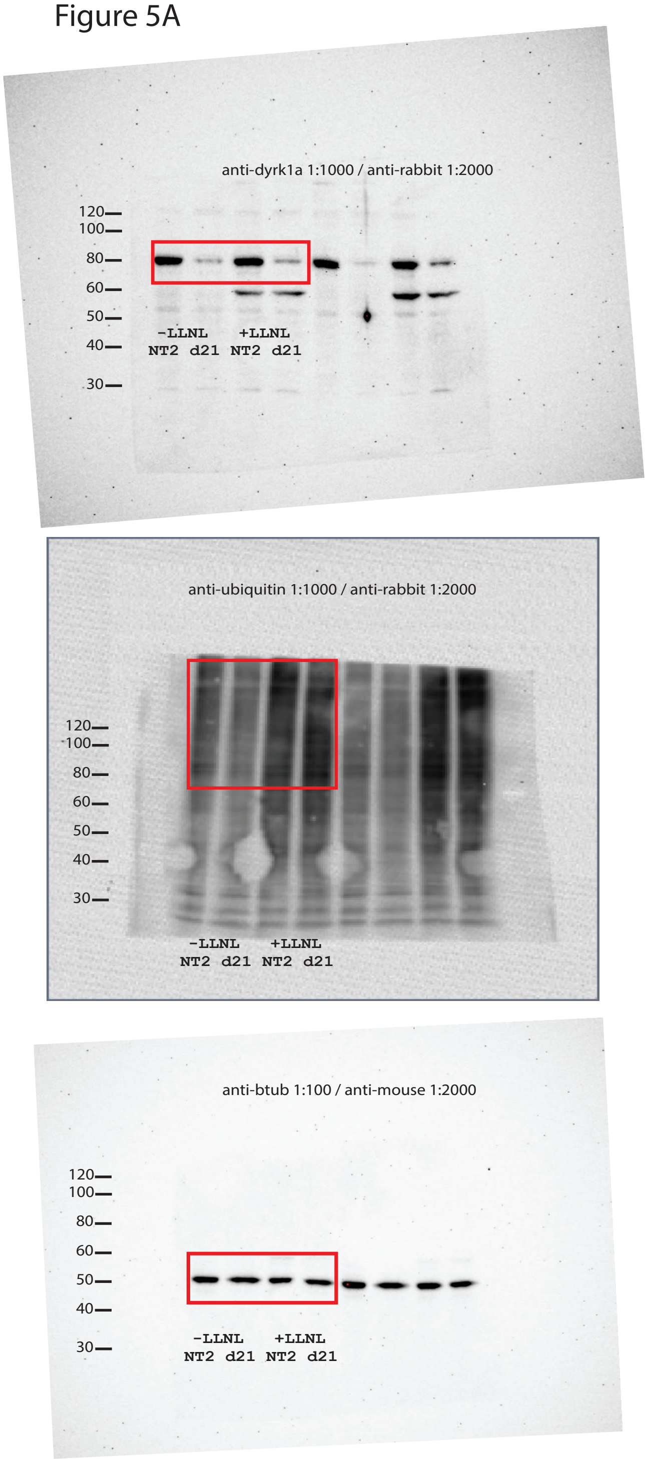

Figure 5C (same blot from 2A right)

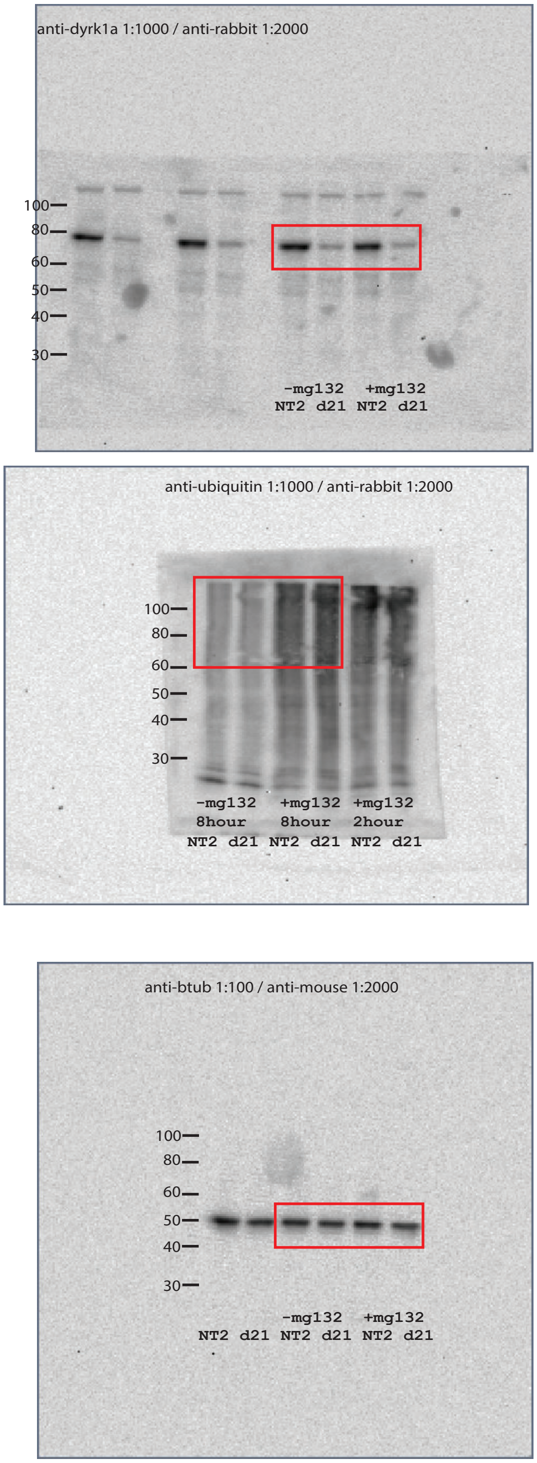

Figure 6B

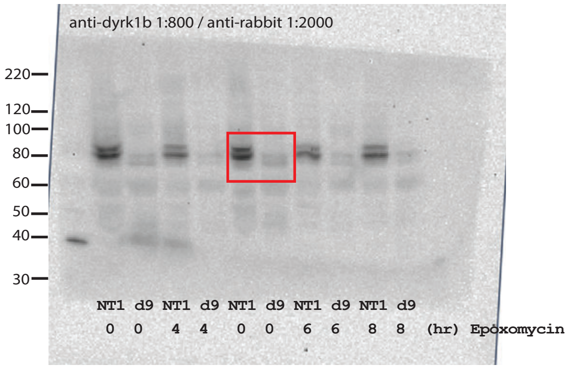
anti-dyrk1b 1:800 / anti-rabbit 1:2000
220
120
100
80
60
50
40
30
NT1 d9 NT1 d9 NT1 d9 NT1 d9 NT1 d9
0 0 4 4 0 0 6 6 8 8 (hr) Epoxomycin

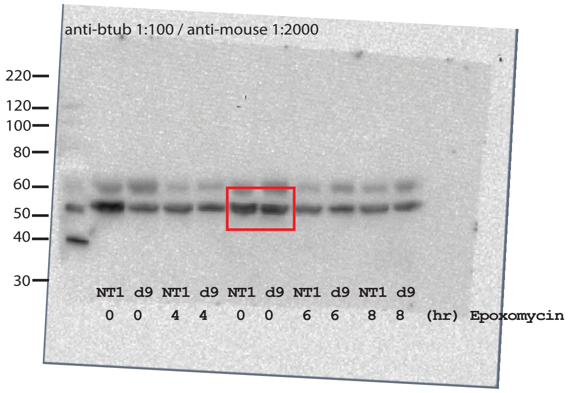
anti-btub 1:100 / anti-mouse 1:2000
220
120
100
80
60
50
40
30
NT1 d9 NT1 d9 NT1 d9 NT1 d9 NT1 d9
0 0 4 4 0 0 6 6 8 8 (hr) Epoxomycin

Figure 7E

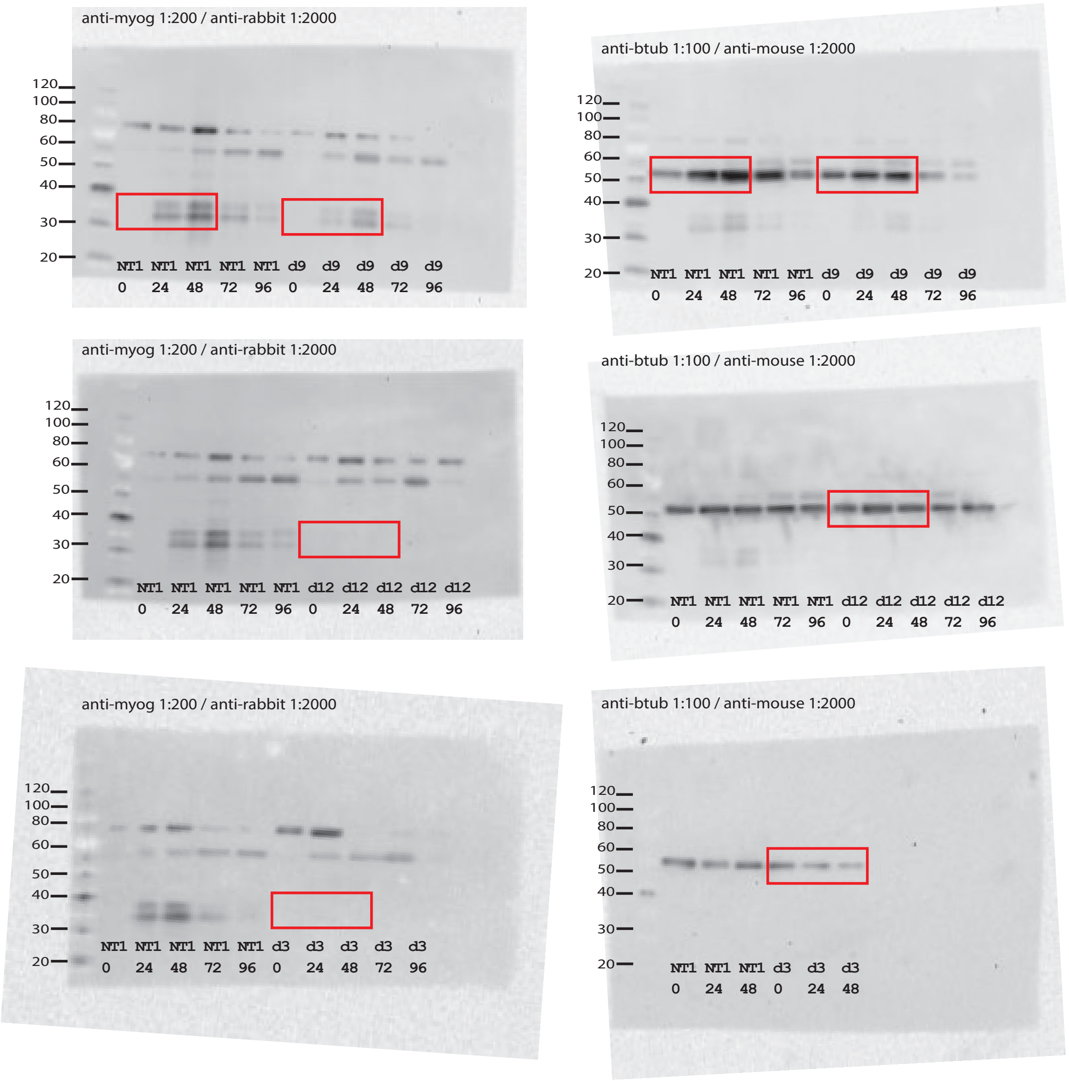

anti-myog 1:200 / anti-rabbit 1:2000
120
100
80
60
50
40
30
20
NT1 NT1 NT1 NT1 NT1 d9 d9 d9 d9 d9
0 24 48 72 96 0 24 48 72 96
anti-btub 1:100 / anti-mouse 1:2000
NT1 NT1 NT1 NT1 NT1 d9 d9 d9 d9 d9
0 24 48 72 96 0 24 48 72 96
anti-myog 1:200 / anti-rabbit 1:2000
NT1 NT1 NT1 NT1 NT1 d12 d12 d12 d12 d12
0 24 48 72 96 0 24 48 72 96
anti-btub 1:100 / anti-mouse 1:2000
NT1 NT1 NT1 NT1 NT1 d12 d12 d12 d12 d12
0 24 48 72 96 0 24 48 72 96
anti-myog 1:200 / anti-rabbit 1:2000
NT1 NT1 NT1 NT1 NT1 d3 d3 d3 d3 d3
0 24 48 72 96 0 24 48 72 96
anti-btub 1:100 / anti-mouse 1:2000
NT1 NT1 NT1 d3 d3 d3
0 24 48 0 24 48

S1A Figure

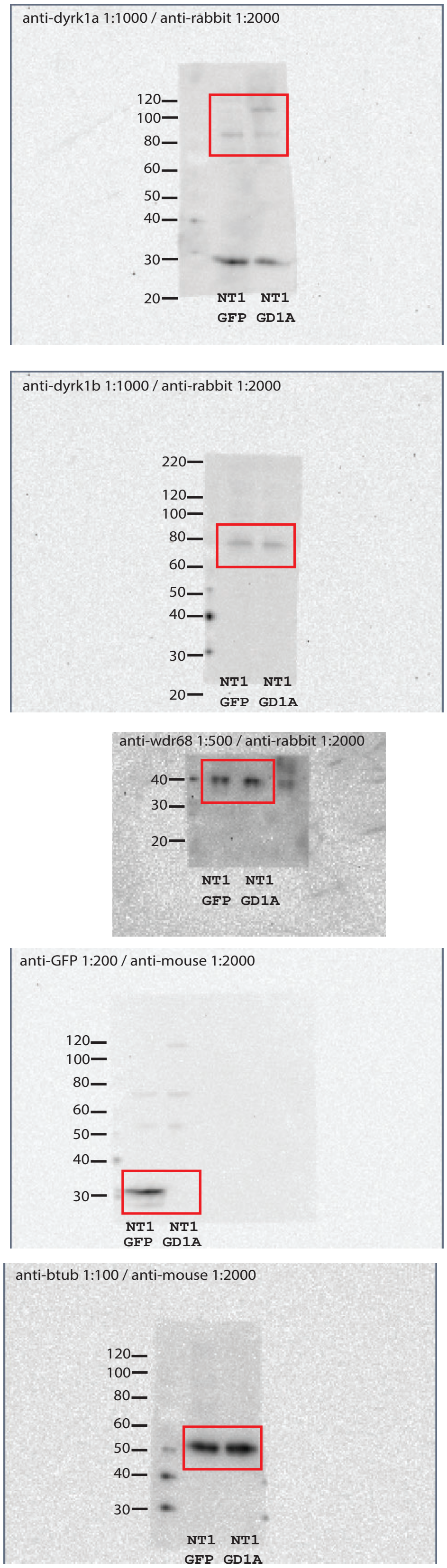

S1B Figure

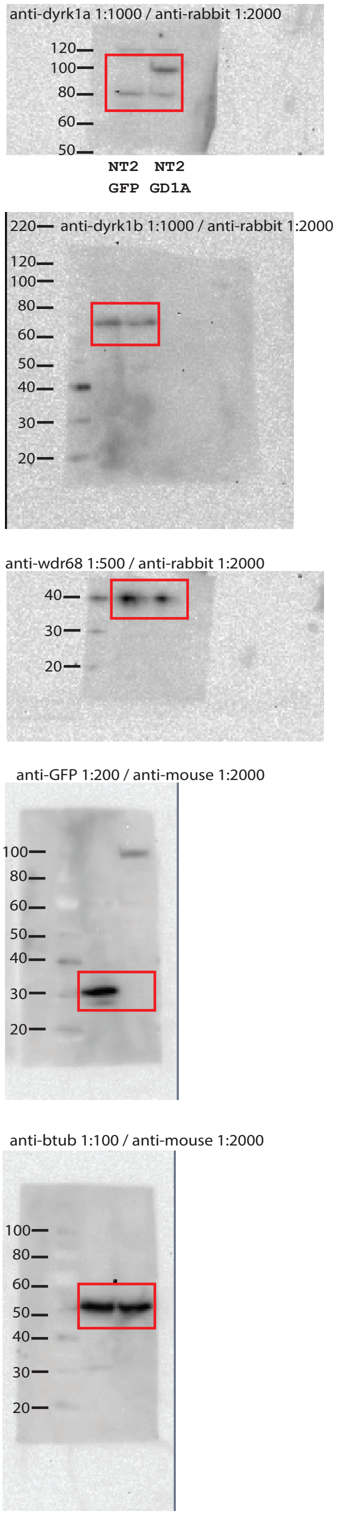

S2A Figure

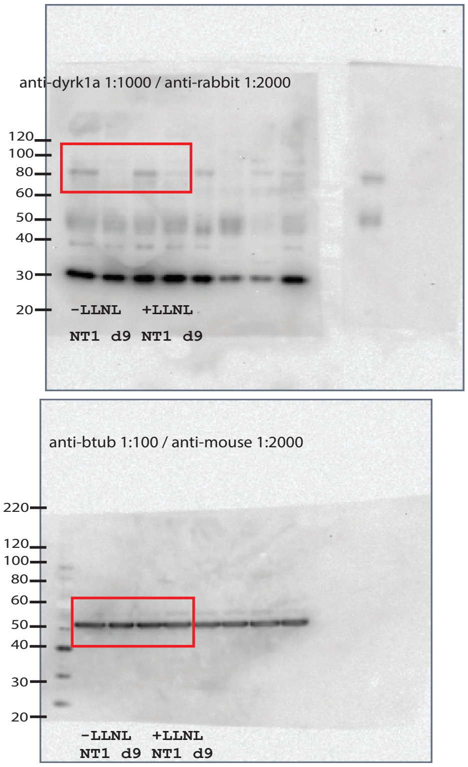

S2B Figure

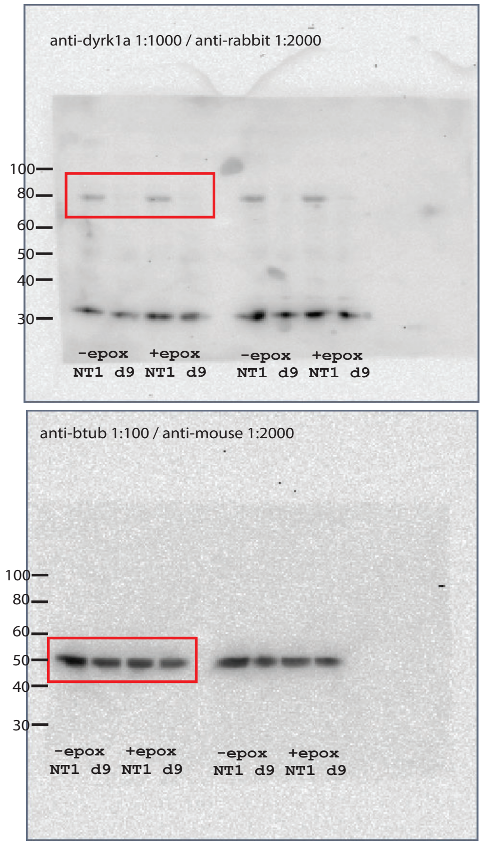

S2C Figure

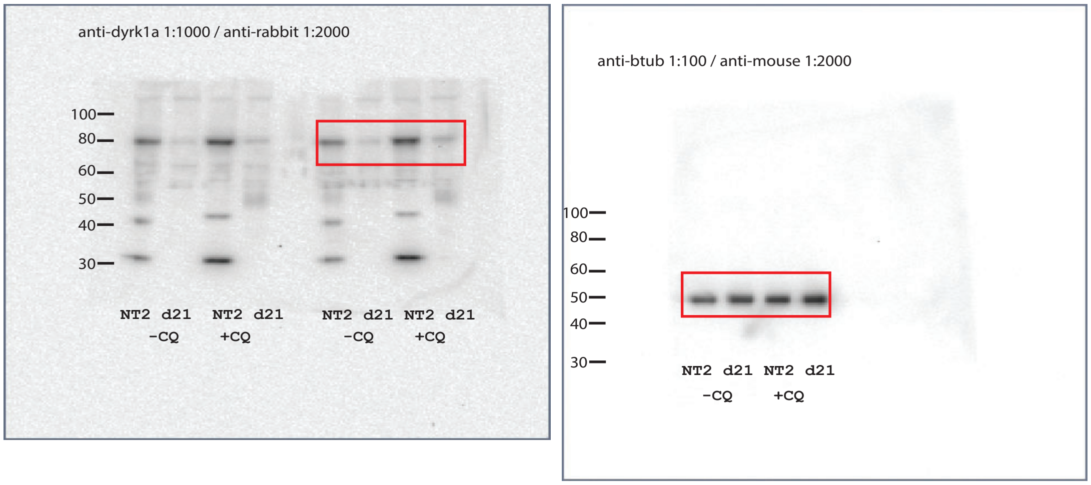

S3A Figure

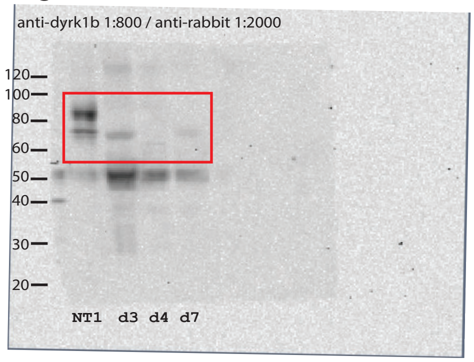
anti-dyrk1b 1:800 / anti-rabbit 1:2000
120
100
80
60
50
40
30
20
NT1 d3 d4 d7

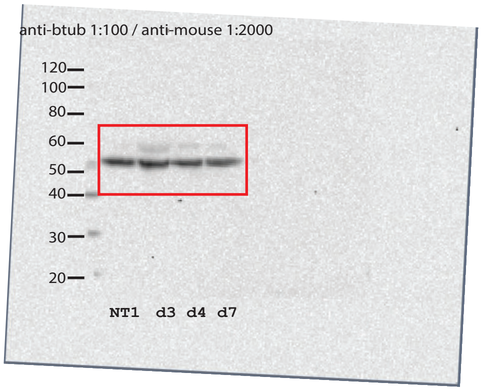
anti-btub 1:100 / anti-mouse 1:2000
120
100
80
60
50
40
30
20
NT1 d3 d4 d7

S4 Figure

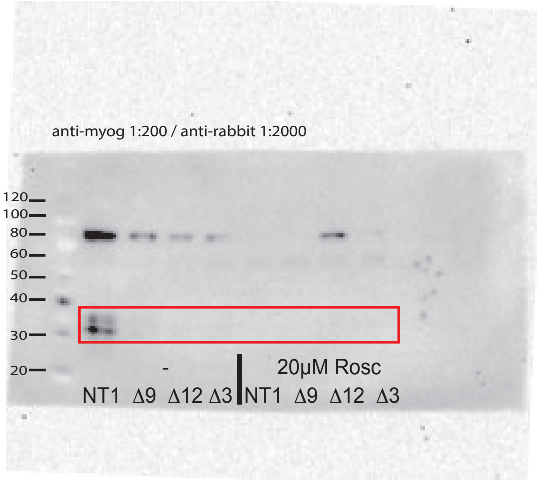
anti-myog 1:200 / anti-rabbit 1:2000
120
100
80
60
50
40
30
20
-
20μM Rosc
NT1 Δ9 Δ12 Δ3 NT1 Δ9 Δ12 Δ3

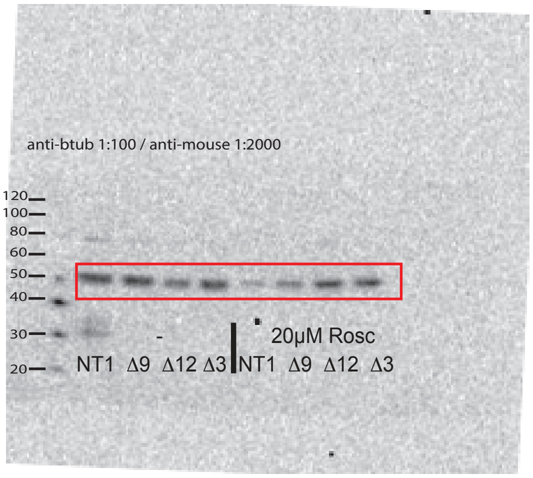
anti-btub 1:100 / anti-mouse 1:2000
120
100
80
60
50
40
30
20
-
20μM Rosc
NT1 Δ9 Δ12 Δ3 NT1 Δ9 Δ12 Δ3
